# Soy Product Consumption and the Risk of Cancer: A Systematic Review and Meta-Analysis of Observational Studies

**DOI:** 10.3390/nu16070986

**Published:** 2024-03-28

**Authors:** Chenting Wang, Keqing Ding, Xuanzhen Xie, Jinyue Zhou, Pengju Liu, Shuang Wang, Ting Fang, Guozhang Xu, Chunlan Tang, Hang Hong

**Affiliations:** 1 School of Public Health, Health Science Center, Ningbo University, Ningbo 315211, China; wangchenting1@163.com (C.W.); zesyunzan@163.com (X.X.); jinyuez0129@163.com (J.Z.); 13596266939@163.com (P.L.); nianniancat@foxmail.com (S.W.); fangting@nbu.edu.cn (T.F.); 2 Ningbo Center for Disease Control and Prevention, Ningbo 315010, China; dingkeqin@163.com

**Keywords:** soy product, cancer, meta-analysis, dose–response, observational study

## Abstract

Background: The association between soy product consumption and cancer risk varies among studies. Therefore, this comprehensive meta-analysis of observational studies examines the association between soy product consumption and total cancer risk. Methods: This study was conducted following the PRISMA guidelines. Up to October 2023, all eligible published studies were searched through PubMed and Web of Science databases. Results: A total of 52 studies on soy product consumption were included in this meta-analysis (17 cohort studies and 35 case–control studies). High consumption of total soy products (RR: 0.69; 95% CI: 0.60, 0.80), tofu (RR: 0.78; 95% CI: 0.70, 0.86), and soymilk (RR: 0.75; 95% CI: 0.60, 0.93) were associated with reduced total cancer risk. No association was found between high consumption of fermented soy products (RR: 1.18; 95% CI: 0.95, 1.47), non-fermented soy products (RR: 0.95; 95% CI: 0.77, 1.18), soy paste (RR: 1.00; 95% CI: 0.88, 1.14), miso soup (RR: 0.99; 95% CI: 0.87, 1.12), or natto (RR: 0.96; 95% CI: 0.82, 1.11) and cancer risk. A 54 g per day increment of total soy products reduced cancer risk by 11%, a 61 g per day increment of tofu reduced cancer risk by 12%, and a 23 g per day increment of soymilk reduced cancer risk by 28%, while none of the other soy products were associated with cancer risk. Conclusion: Our findings suggest that high total soy product consumption, especially soymilk and tofu, is associated with lower cancer risk. More prospective cohort studies are still needed to confirm the causal relationship between soy product consumption and cancer risk.

## 1. Introduction

The incidence of cancer is rising dramatically, and it is the leading cause of death worldwide. There were nearly 19.3 million new cancer cases and 10 million deaths worldwide in 2020, according to the GLOBOCAN database. The cancer with the highest number of new cases is breast cancer, followed by lung cancer, colorectal cancer, prostate cancer, and stomach cancer, and these cancers are also the leading causes of cancer deaths [1]. Breast, prostate, and colorectal cancers are lower in Asia than in the Western regions, while the stomach and esophagus cancers are very common, which may be related to different regional lifestyles and dietary habits [1,2,3,4,5]. Soy products are processed foods made from beans as the primary raw material, and as one of the main food items for Asian populations, soy products are consumed more in Asia than in the West [6,7]. As a valuable source of isoflavones, phytosterols, lecithin, polyunsaturated fatty acid, dietary fiber, and high-quality protein, soy products have attracted considerable attention for their potential to reduce the risk of cancer [8]. Isoflavones can inhibit tumor growth and induce apoptosis in cancer cells through pathways mediated by hormone and non-hormone receptors [9,10,11]. Given the different production processes of soy products, the effects of varying soy products on cancer may not be the same. The results of several epidemiological studies support these ideas, such as the findings that tofu may reduce the risk of gastrointestinal cancer [12,13], while soy paste may increase this risk [5]. In addition, the same soy product may even have different effects on different types of cancer. Excessive soy paste intake may reduce the risk of breast cancer [14], but it may also increase the risk of colorectal cancer [15]. At the same time, the relationship between soy product consumption and cancer risk can be observed differently after considering specific characteristics of the participants, such as gender and country [16,17,18,19,20].

Previous meta-analyses have analyzed the association between soy products and cancer, either for one kind of soy product or one particular type of disease. Wang et al. [21] studied the relationship between fermented and non-fermented soy product consumption and the risk of gastric cancer. Another meta-analysis focused on the association between tofu consumption and breast cancer risk [22]. Although Woo et al. [23] examined the relationship between one type of soy product consumption and the risk of several cancers, the study was not explicitly designed to address the soy product and cancer risk hypothesis and did not provide an overall estimate of total cancer risk. In addition, previous meta-analyses have given inconsistent conclusions about the association between soy products and the risk of breast and gastrointestinal cancers [24,25,26,27,28,29,30]. This may be related to the types of soy products included in different studies and the different definitions of high exposure, so it is necessary to figure out which soy products are health-protective and to assess their effect quantitatively. In conclusion, there is no comprehensive meta-analyses on the influence of soy product consumption on cancer risk.

The purpose of this study was to systematically review the association between soy products (including total soy products, fermented soy products, non-fermented soy products, tofu, soymilk, soy paste, miso soup, and natto) and cancer risk in observational studies, conducting a comprehensive meta-analysis to provide an overall estimate of total cancer risk. Furthermore, a dose–response meta-analysis was carried out to quantitatively assess soy products’ role in cancer.

## 2. Materials and Methods

### 2.1. Literature Search

The systematic review and meta-analysis was registered (PROSPERO ID: CRD42023466077), and this study was conducted following the PRISMA guidelines [31] (Supplemental Table S1). A systematic literature search for studies was performed by two independent authors using the databases PubMed and Web of Science until October 2023 with the following keywords and their synonyms: “(soy OR bean OR soybean OR isoflavones OR isoflavone OR soy isoflavones OR soy products OR phytoestrogen OR daidzein OR glycitein OR genistein OR soy protein OR tofu OR soy foods OR tempeh OR soya OR sufu OR glycine max OR bean curd OR soymilk OR miso OR pea OR Legume OR lentil OR natto) AND (neoplasms OR neoplasm OR neoplasia OR neoplasias OR cancer OR cancers OR carcinoma OR tumor OR tumour)”.

### 2.2. Study Selection and Exclusion Criteria

**Figure 1:**
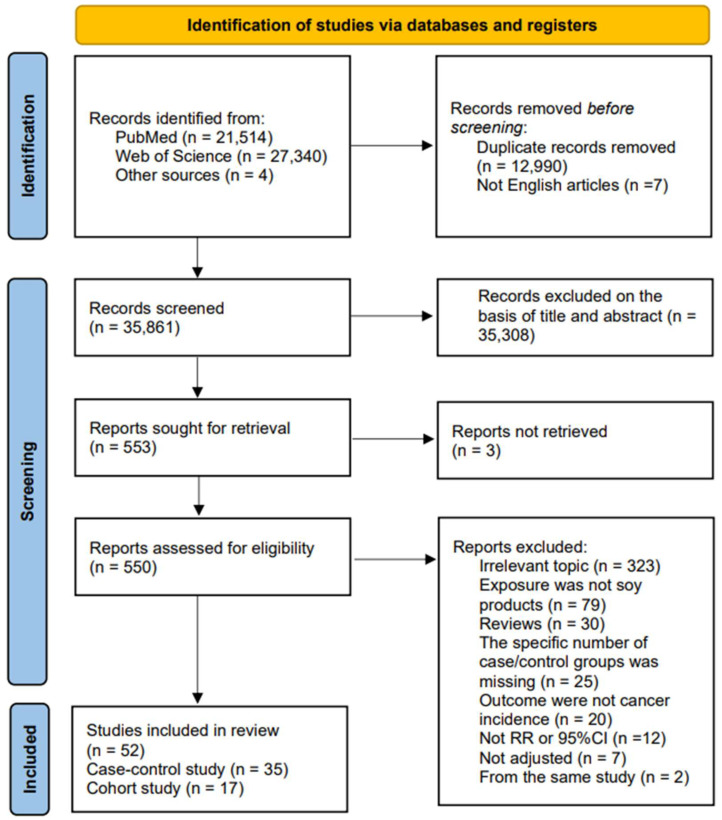
Flow diagram of the literature search and study selection.

The inclusion criteria were as follows: (1) case–control or cohort studies; (2) studies that reported the specific number of cases and participants in each category; (3) studies that evaluated the association between the consumption of soy foods and the risk of cancer; (4) studies that presented adjusted odds ratio (OR), relative risk (RR), or hazard ratio (HR), as well as 95% confidence intervals (95% CI). Review articles, letters, animal research articles, and a range of other studies that could not be used for statistical analysis, as well as non-English studies, were excluded. If there was more than one article from the same study, the latest one was selected. The detailed process of study selection is shown in Figure 1.

### 2.3. Date Extraction and Quality Assessment

Two authors independently extracted the following data from the included studies: (1) the first author’s name and the year of publication; (2) country and study name; (3) study design; (4) study period; (5) age of subjects; (6) the number of cases and participants; (7) each category of exposure consumption; (8) the type of cancer; (9) adjusted covariates; (10) adjusted OR/HR/RR and 95% CI. The most adjusted one was extracted when a study reported several OR, HR, and RR. The quality of cohort and case–control studies included in the meta-analysis was assessed using the Newcastle–Ottawa Scale [32]. An article is deemed to be of exceptional quality if its score surpasses 7. Conversely, it is considered inferior if its score falls below this threshold (out of 9).

### 2.4. Statistical Analysis

The pooled adjusted RRs and their 95% CIs of cancer risk for the highest compared with the lowest consumption categories, comprising total soy products, tofu, miso soup, fermented soy products, soymilk, soy paste, natto, and non-fermented, was used to assess the effect of high consumption of these soy products on cancer risk using the DerSimonian and Laird random effects model, which considers both within-study and between-study variations [33]. ORs and HRs were considered equivalent to RRs. If a study reported separately by gender [15,16,17,18,20,34,35,36,37,38,39,40,41], different ages [42,43], menopausal status [14,44], smoking [45], cancer type [46], EGFR mutation [16], or BRCA mutation [47], the overall estimate was obtained by the fixed-effect model before merging them with other studies. Subgroup analysis was conducted by cancer type (gastrointestinal cancer, gynecological cancer, upper aerodigestive tract cancer, prostate cancer, lung cancer, bladder cancer, liver cancer, multiple myeloma, non-Hodgkin lymphoma, and leukemia). Subgroup analyses also stratified the data by sex, study design (case–control study, cohort study), and geographic location (China, Japan, Korea, Singapore, Europe, USA). Meta-regression analysis was used to explore possible heterogeneity between studies further. Statistical heterogeneity among studies was determined using the Q test and *I*^2^ statistic. *I*^2^ values > 50% were considered high heterogeneity [48].

A study could be included in the dose–response meta-analysis when it provided adjusted RRs and 95% CIs with at least three exposure categories and the number of person-years, cases, and participants for each exposure category. Linear or nonlinear dose–response meta-analysis was conducted using the methods proposed by Greenland, Longnecker [49] and Orsini et al. [50,51]. For the linear relationship between soy product consumption and total cancer risk, a 2-stage dose–response meta-analysis was used. The nonlinear dose–response model was established using restricted cubic splines with 3 knots at 10%, 50%, and 90% percentiles of the distribution. Random or fixed effects models were selected according to the size of heterogeneity and the degree of model fit. Median exposure consumption was considered the value of the assigned dose. If a study provided upper and lower boundaries, the midpoint value was selected as the given dose. For the open-ended exposure categories, adjacent categories were assumed to have the same interval. In addition, the average consumption was considered as the given dose if the study reported only the average consumption.

Potential publication bias was detected by Begg [52] and Egger tests [53]. In addition, the trim-and-fill method was used to test and adjust the effect of potential publication bias on the results [54]. Moreover, a sensitivity analysis was conducted to assess the stability of our results. The linear or nonlinear trends were assessed by the Wald test [55]. A *p*-value < 0.05 was considered statistically significant for all analyses. All statistical analyses were performed by using Stata 17.0 (Stata Corp) and R 4.3.2 (R Foundation for Statistical Computing, Vienna, Austria)

## 3. Result

### 3.1. Study Characteristics

After the layers of screening, a total of 52 studies (35 articles were case–control studies [11,12,13,14,15,16,18,38,39,41,42,43,44,45,46,56,57,58,59,60,61,62,63,64,65,66,67,68,69,70,71,72,73,74,75] and 17 articles were cohort studies [17,19,20,34,35,36,37,40,47,76,77,78,79,80,81,82,83]) were included in this meta-analysis, with 861,372 participants and 44,932 cases. The characteristics of the included studies are shown in Table 1. Regarding geographic location, 42 articles reported data from Asia (16 from Japan, 14 from China, 9 from Korea, and 3 from Singapore), 8 articles reported data from America, and the last 2 were from Europe. Regarding quality assessment, the case–control studies achieved an average score of 6.7. The mean score of the cohort study was 7.2, which satisfied the criterion of high quality. All analyses adjusted for age, and most studies adjusted for smoking status (n = 38), drinking status (n = 29), total energy intake (n = 27), BMI (n = 26), and education level (n = 26) (Supplemental Tables S2 and S3).

### 3.2. Total Soy Product Consumption and Cancer Risk

**Figure 2:**
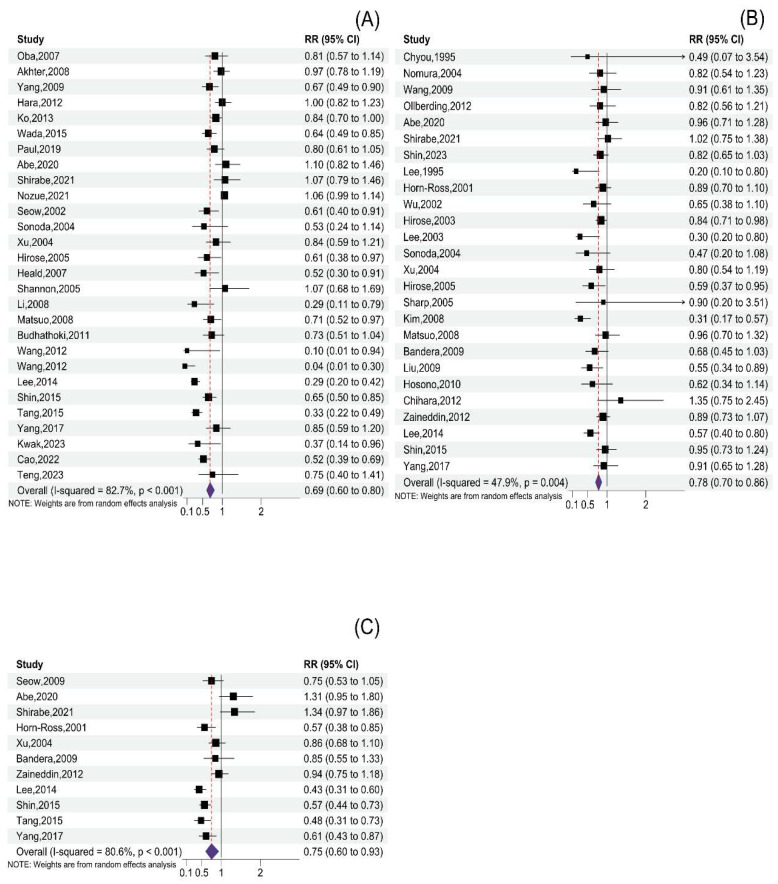
Forest plot of cancer risk for the highest versus lowest categories of soy product consumption: (**A**) Forest plot of cancer risk for the highest versus lowest categories of total soy product consumption. Data is from references [14,15,16,18,19,20,35,36,37,39,40,41,45,47,60,61,63,64,66,69,70,72,73,74,75,80,82,83]. (**B**) Forest plot of cancer risk for the highest versus lowest categories of tofu consumption. Data is from references [12,13,14,15,16,17,18,20,38,44,46,56,57,60,61,62,65,67,68,71,72,76,77,79,81,83]. (**C**) Forest plot of cancer risk for the highest versus lowest categories of soymilk consumption. Data is from references [15,18,20,56,61,67,71,72,73,78,83].

**Figure 3:**
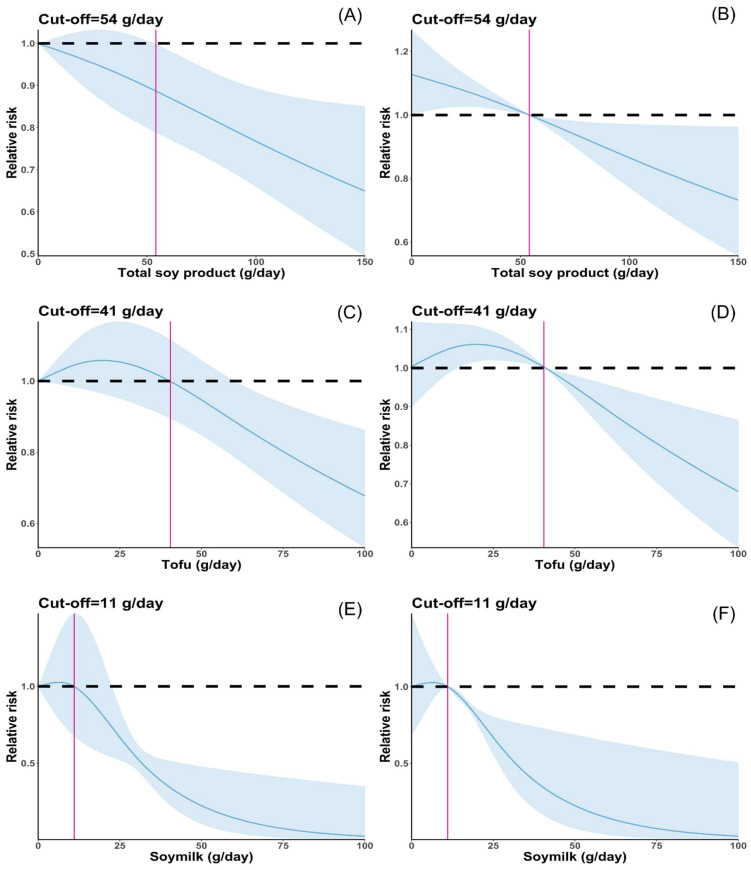
Dose–response analysis of soy product and the risk of cancer (**A**) Dose–response study of total soy product and cancer risk. (**B**) Dose–response analysis of total soy product and cancer risk, with reference dose as cut-off point. (**C**) Dose–response analysis of tofu and the risk of cancer. (**D**) Dose–response analysis of tofu and cancer risk, with reference dose as cut-off point. (**E**) Dose–response analysis of soymilk and the risk of cancer. (**F**) Dose–response analysis of total soymilk and the risk of cancer, with reference dose as cut-off point. Relative risks are indicated by solid lines, the blue-shaded regions indicate the 95% confidence intervals, and purple vertical line indicate the reference point.

A total of 28 studies (18 case–control studies and 10 cohort studies) evaluated the relationship between total soy product consumption and cancer risk, with 553,282 participants and 24,090 cases. Compared with the lowest total soy product consumption of subjects, cancer risk of the highest total soy product consumption was reduced by 31% (RR: 0.69; 95% CI: 0.60; 0.80) in the pooled estimates, with highly significant heterogeneity among the studies (*I*^2^ = 82.7%, *p* < 0.001) (Figure 2A, Table 2, Supplemental Table S4). The association between the highest soy product consumption and cancer risk was observed in case–control studies (RR: 0.56; 95% CI: 0.46, 0.69) but not in cohort studies (RR: 0.9; 95% CI: 0.80, 1.01) and the type of study design was a source of heterogeneity (*p*-difference = 0.004). Women who consume high quantities of soy products have a 24% reduced risk of cancer (RR: 0.76; 95% CI: 0.65, 0.89). Notably, no such association was observed in the male cohort of the study (RR: 0.86; 95% CI: 0.74, 1.00). Interestingly, no significant difference between the sexes was observed (*p*-difference = 0.454). For the cancer type, the associations were discovered in gastrointestinal cancer (RR: 0.74; 95% CI: 0.61, 0.89), prostate cancer (RR: 0.47; 95% CI: 0.31, 0.71), lung cancer (RR: 0.67; 95% CI: 0.52, 0.86), upper aerodigestive tract cancer (RR: 0.33; 95% CI: 0.22, 0.49), and multiple myeloma (RR: 0.10; 95% CI: 0.01, 0.97), but not in bladder or liver cancer. From an extensive analysis of gynecological cancers, consuming high amounts of soy products may decrease cancer risk (RR: 0.71; 95% CI: 0.54, 0.92). However, upon conducting separate analyses of the four types of gynecological cancer, these inverse associations were only present in ovarian cancer (RR: 0.29; 95% CI: 0.20, 0.42). Only one article reported these associations (*p*-difference > 0.05 for all comparisons). According to the geographic location, the risk of cancer was found to be lower in Korea (RR: 0.75; 95% CI: 0.61, 0.92), Singapore (RR: 0.73; 95% CI: 0.57, 0.94), Europe (RR: 0.52; 95% CI: 0.60, 0.80), and China (RR: 0.48; 95% CI: 0.34, 0.69). However, no such associations were found in the USA (RR: 0.73; 95% CI: 0.51, 1.04) or Japan (RR: 0.89; 95% CI: 0.77, 1.02). Furthermore, the meta-regression analysis results showed a statistical difference between China and Japan (*p*-difference = 0.022). Twenty-five studies [15,16,18,19,20,35,36,37,39,40,41,60,61,72,73,74,80,83] were included in the dose–response analysis for total soy product consumption and risk of cancer, and there was a nonlinear relationship between them (*p*-nonlinear = 0.0028) (Figure 3A,B). When an additional 54 g of total soy product was consumed daily, the cancer risk began to decline (RR: 0.89; 95% CI: 0.79, 0.99). Consuming an additional 100 and 150 g of total soy products per day reduced cancer risk by 23% (RR: 0.77; 95% CI: 0.66, 0.89) and 35% (RR: 0.65; 95% CI: 0.50, 0.85), respectively.

### 3.3. Tofu Consumption and Cancer Risk

Nineteen case–control studies and seven cohort studies, including 312,770 participants and 18,729 cases, investigated the relationship between tofu consumption and cancer risk. In the pooled estimates, cancer risk was significantly reduced in subjects with the highest tofu consumption compared to those with the lowest tofu consumption (RR: 0.78; 95% CI: 0.70, 0.86), and heterogeneity among the studies was low (*I*^2^ = 47.9%, *p* = 0.004) (Figure 2B, Table 2). High tofu consumption reduced cancer risk in both men and women. In addition, a significant association was detected in case–control studies (RR: 0.72; 95% CI: 0.63, 0.83) but not in cohort studies (RR: 0.89; 95% CI: 0.78, 1.01) (*p*-difference = 0.186). As for cancer type, the association was not detected in liver cancer, prostate cancer, lung cancer, non-Hodgkin lymphoma, or upper aerodigestive tract cancer. Tofu consumption has been linked to gastrointestinal (RR: 0.67; 95% CI: 0.47, 0.96) and gynecological cancers (RR: 0.76; 95% CI: 0.66, 0.87), particularly associated with stomach (RR: 0.56; 95% CI: 0.34, 0.93), breast (RR: 0.79; 95% CI: 0.66, 0.94), endometrial (RR: 0.77; 95% CI: 0.61, 0.97), and ovarian cancers (RR: 0.57; 95% CI: 0.40, 0.81). Conversely, no such anti-cancer effect was found in colorectal (RR: 0.95; 95% CI: 0.73, 1.24) or cervical cancer (RR: 0.62; 95% CI: 0.34, 1.14), which may be related to the number of studies (n = 1, respectively). In addition, one study found that tofu consumption may reduce the risk of leukemia (RR: 0.55; 95% CI: 0.34, 0.89). However, there was no statistical difference by cancer type in meta-regression analysis (*p*-difference > 0.05 for all comparisons). By geographic location, a significant inverse association was shown in the USA (RR: 0.82; 95% CI: 0.71, 0.95), Korea (RR: 0.58; 95% CI: 0.40, 0.85), and China (RR: 0.63; 95% CI: 0.50, 0.80), but could not be found in Japan (RR: 0.87; 95% CI: 0.76, 1.00) or Europe (RR: 0.89; 95% CI: 0.74, 1.08). In addition, a significant difference between Japan and China was observed (*p*-difference = 0.049). Twelve studies [15,18,20,60,61,62,65,72,77,83] were included in the dose–response analysis for tofu consumption and risk of cancer risk, and the results showed a nonlinear relationship between them (*p* for nonlinear = 0.0055) (Figure 3C,D). Cancer risk began to decline when tofu consumption increased by 61 g daily (RR: 0.88; 95% CI: 0.78, 0.99). Increased consumption of 100 g of tofu per day was associated with a 32% reduction in cancer risk (RR: 0.68; 95% CI: 0.53, 0.86).

### 3.4. Soymilk Consumption and Cancer Risk

A total of 8269 cases and 177,626 participants in eleven studies (eight case–control studies and three cohort studies) reported soymilk consumption. In the pooled estimates, high consumption of soymilk was inversely associated with cancer risk (the highest versus the lowest category) (RR: 0.75; 95% CI: 0.60, 0.93), and the heterogeneity among studies was high (*I*^2^ = 80.6%, *p* < 0.001) (Figure 2C, Table 2). In case–control studies, increased consumption of soymilk showed an inverse association with cancer risk (RR: 0.65; 95% CI: 0.52, 0.80), but no association was found in cohort studies (RR: 1.10; 95% CI: 0.76, 1.58), and the type of study design was a source of heterogeneity (*p*-difference = 0.031). According to the sex stratification, no significant association was found (*p*-difference = 0.699). By cancer type, significant associations were detected for gastrointestinal cancer (RR: 0.58; 95% CI: 0.47, 0.72), ovarian cancer (RR: 0.43; 95% CI: 0.31, 0.6), and upper aerodigestive cancer (RR: 0.48; 95% CI: 0.31, 0.74), but not for gynecological cancer (breast and endometrial), lung cancer, or liver cancer, and there was no statistical difference in meta-regression analysis (*p*-difference > 0.05 for all comparisons). Based on geographic location, soymilk consumption was found to be inversely associated with cancer risk in China (RR: 0.57; 95% CI: 0.35, 0.93) and Korea (RR: 0.58; 95% CI: 0.47, 0.72), while not in the USA, Singapore, or Europe. In contrast, soymilk consumption showed a positive association with cancer in Japan (RR:1.32; 95% CI:1.05, 1.66). However, a statistical difference was only observed between Korea and Japan (*p*-difference = 0.035), but not between China and Japan (*p*-different = 0.068). Six studies [15,61,72,73] were included in the dose–response between soymilk consumption and cancer risk, and there was a nonlinear relationship between them (*p* for nonlinear < 0.001) (Figure 3E,F). When an additional 23 g of total soy product was consumed daily, the cancer risk began to decline (RR: 0.72; 95% CI: 0.54, 0.99). Increased consumption of 30 g of soymilk per day was associated with a 46% reduction in cancer risk (RR: 0.54; 95% CI: 0.46, 0.63).

### 3.5. Other Soy Product Consumption and Cancer Risk

**Figure 4:**
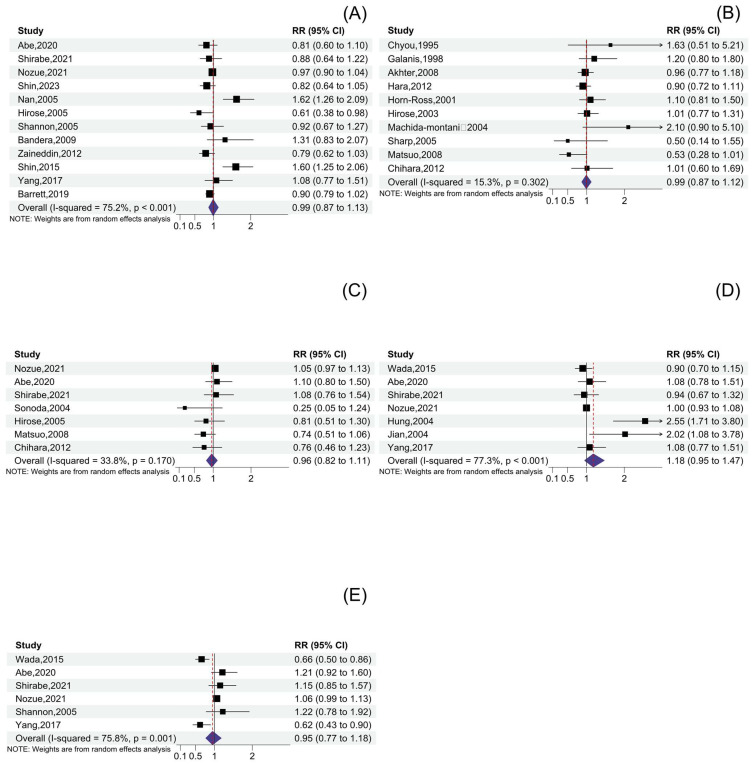
Forest plot of cancer risk for the highest versus lowest categories of soy product consumption: (**A**) Forest plot of cancer risk for the highest versus lowest categories of soy paste consumption. Data is from references [5,14,15,17,18,20,37,43,63,67,71,83]. (**B**) Forest plot of cancer risk for the highest versus lowest categories of miso soup consumption. Data is from references [16,34,35,36,38,44,56,59,62,76]. (**C**) Forest plot of cancer risk for the highest versus lowest categories of natto consumption. Data is from references [16,20,37,38,44,83]. (**D**) Forest plot of cancer risk for the highest versus lowest categories of fermented soy product consumption. Data is from references [18,19,20,37,42,58,83]. (**E**) Forest plot of cancer risk for the highest versus lowest categories of non-fermented soy product consumption. Data is from references [18,19,20,37,63,83].

**Figure 5:**
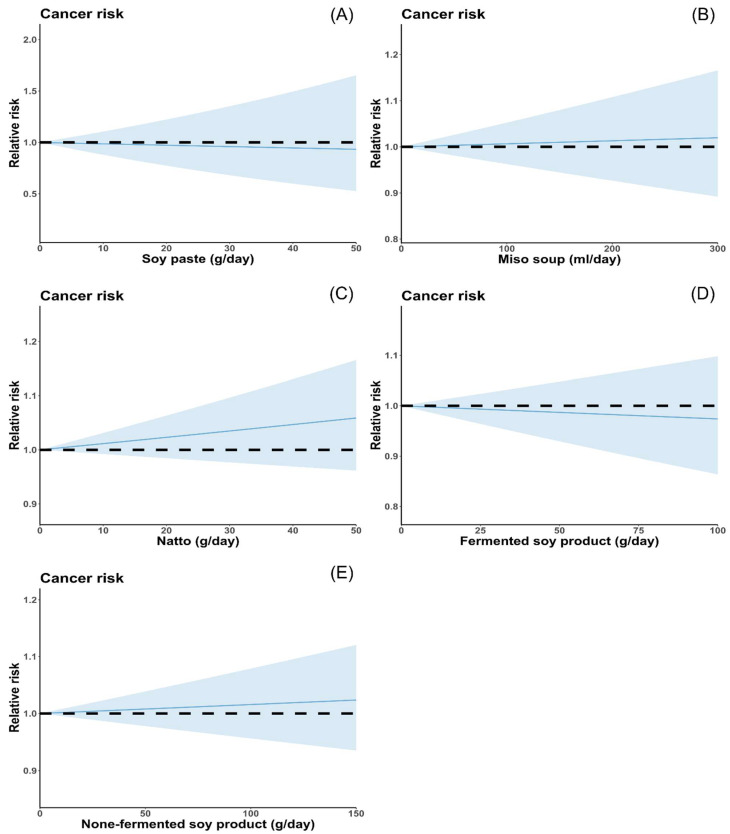
Dose–response analysis of soy product and the risk of cancer: (**A**) Dose–response study of soy paste and cancer risk. (**B**) Dose–response analysis of natto and the risk of cancer. (**C**) Dose–response analysis of miso soup and the risk of cancer. (**D**) Dose–response analysis of fermented soy product and the risk of cancer. (**E**) Dose–response analysis of non-fermented soy product and the risk of cancer. Relative risks are indicated by solid lines, and the blue-shaded regions indicate the 95% confidence intervals.

A total of twelve soy paste studies, ten miso soup studies, seven natto studies, seven fermented soy product studies, and six non-fermented soy product studies were included in this meta-analysis. The pooled RRs for the highest consumption of soy paste, miso soup, natto, fermented soy products, and non-fermented soy products versus the lowest consumption categories were 0.99 (95% CI: 0.87, 1.13), 0.99 (95% CI: 0.87, 1.12), 0.96 (95% CI: 0.82, 1.11), 1.18 (95% CI: 0.95, 1.47), and 0.95 (95% CI: 0.77, 1.18) (Figure 4, Table 3). There was heterogeneity between studies on soy paste, fermented, and non-fermented soy products, but no heterogeneity between natto and miso soup studies. Although these soy products were not associated with a reduced risk of total cancer, the results were changed upon conducting a subgroup analysis. In terms of study type, the pooled results of the four case–control studies showed that high consumption of natto was associated with a reduced risk of total cancer (RR: 0.74; 95% CI: 0.58, 0.95), and the meta-regression results suggested that the study design might be the source of heterogeneity in natto studies (*p*-difference = 0.045). For cancer types, consuming large amounts of non-fermented soy products reduces the risk of gastric cancer, while consuming a lot of soy paste reduces the risk of breast cancer. Regarding geographic location, the pooled result from studies in China found that high consumption of fermented soy products may be associated with an increased risk of cancer. Finally, no significant difference was found in total cancer risk between men and women (*p*-difference > 0.05 for all comparisons). In the dose–response meta-analysis, all of these soy products showed a linear relationship with cancer risk (*p* for nonlinear = 0.48 for soy paste, 0.77 for miso soup, 0.19 for natto, 0.20 for fermented soy products, and 0.84 for non-fermented soy products), and no associations were found between high consumption of these soy products and cancer risk (Figure 5).

### 3.6. Sensitivity Analysis and Publication Bias

Sensitivity analysis was conducted by systematically removing one study at a time and combining the remaining studies for meta-analysis. After analyzing the mixed results, the meta-analysis results did not change due to the influence of certain studies. Egger and Begg tests were adopted to detect publication bias, and no obvious publication bias was found in miso soup, fermented soy food, non-fermented soy food, soymilk, or paste (all *p* ≥ 0.05). However, some evidence of publication bias was found in total soy food (Egger test *p* < 0.001) and tofu (Egger test *p* = 0.004). Therefore, the trim-and-fill method was further used to evaluate the effect of publication bias on the results. The pooled relative risk (RR) remained unchanged, indicating that the results were authentic and not influenced by publication bias.

## 4. Discussion

Based on 52 observational studies, the present study comprehensively assessed the relationship between consuming various soy products and the risk of cancer. Our study found that high consumption of total soy foods, tofu, and soymilk was associated with a reduced risk of total cancer. The results of the dose–response meta-analysis also supported our findings. Moreover, these adverse correlations were more evident in case–control and Chinese population study subgroup analyses. Nonetheless, no association was found between the high consumption of soy paste, natto, miso soup, fermented soy food, or non-fermented soy food and the risk of total cancer.

To our knowledge, there has been no comprehensive meta-analysis of soy product consumption on total cancer risk. Most previous meta-analyses were conducted to analyze the relationship between soy products and only one type of cancer. Many meta-analyses have found that high consumption of total soy products is associated with a reduced risk of prostate [84,85,86] and lung cancer [87,88,89], which is consistent with our findings. Soy products are a rich source of isoflavones, which are the leading cause of the anti-cancer effects of soy products [90]. Isoflavones are structurally and functionally similar to estrogen, and depending on the concentration of estrogen at each site, isoflavones can bind to become estrogen receptor agonists or antagonists, preventing cancer through estrogen-dependent mechanisms in the estrogen signaling pathway [9,91]. In the process of prostate cancer, the expression of estrogen-β is often lost, and estrogen-β is closely related to the functions of tissue stability and cell proliferation [10,92]. Genistein is one of the essential soy isoflavones that can bind to estrogen-β and inhibit the development of prostate cancer [93]. Similarly, Bogush et al. also found that more than half of breast cancer and lung cancer patients did not express estrogen-β [94]. Previous meta-analyses have found that total soy products were associated with a reduced risk of gastrointestinal cancer [24,25]. However, when gastric cancer and colorectal cancer were analyzed separately, several meta-analyses came to different conclusions [24,95,96,97]. The difference may be related to the types of soy products included in these studies. Most researchers believe that high total soy products may reduce the risk of gastric and colorectal cancer, and our findings further support this view. Isoflavones have anti-inflammatory and antioxidant effects, and they can modulate the NF-kB signaling pathway, which is associated with increased levels of tumor growth factors, especially in gastrointestinal cancer [98,99]. In addition, genistein can induce cytotoxicity in human cancer cells during the G2/M cell cycle phase and reduce cell proliferation by inhibiting cellular topoisomerase [8]. Much controversy still surrounds the relationship between soy products and breast cancer risk [47,74,100]. Although the results of many observational studies have been inconsistent, most past meta-analyses have linked soy product consumption to a reduced risk of breast cancer [26,27,28,29,30]. As far as our findings are concerned, there is no statistically significant risk relationship between total soy products and breast cancer. This may be related to the definition of high consumption and the different types of total soy products included in the studies. Yamamoto et al. [101] suggested that the risk of breast cancer may be related to isoflavone intake rather than total soy products intake, and our study did not mention a focus on isoflavone intake. In addition, Chen et al. [29] pointed out that the results may vary depending on the type of study design. Five studies were included (two cohort studies and three case–control studies). Two of the three case–control studies showed inverse association, while the results of all cohort studies indicated no association. The pooled RR and 95% CI of the two prospective cohort meta-analyses included 1 [27,28]. Future meta-analyses of the association between total soy products and breast cancer risk should include more prospective cohort studies. Moreover, most studies were not explicitly designed to address the soy products and breast cancer hypothesis, which may obscure the genuine relationship [23,26,28].

Our study found an inverse association between high tofu consumption and gastric cancer (RR: 0.56; 95% CI: 0.34, 0.93). However, in the case of colorectal cancer, no such association was found (RR:0.95; 95% CI:0.73, 1.24), which is consistent with previous meta-analyses [23,88,97]. Although the results showed that high tofu consumption can reduce 33% of gastrointestinal cancer (RR: 0.67; 95% CI: 0.47, 0.96), only one study related to colorectal cancer was included in this meta-analysis. Therefore, the association between tofu and gastrointestinal cancers should be interpreted with caution, as the inclusion of additional colorectal cancer studies may change the results. As with previous meta-analyses, the high consumption of tofu was associated with a lower risk of breast cancer [23,30]. A recent meta-analysis examining the relationship between isoflavone-rich food intake and breast cancer failed to find an inverse association between tofu intake and breast cancer, as only two tofu studies were included [27]. Our meta-analysis included only one cohort study, so more prospective studies are necessary to confirm our findings. In terms of prostate cancer, Applegate et al. [84] found an inverse association between tofu intake and prostate cancer risk, while our study did not find such an association (RR: 0.70; 95% CI: 0.43, 1.15). Only two studies were included [60,77], which were included in the previous meta-analysis by Applegate et al. [84]. In addition to this, tofu was inversely associated with endometrial cancer (RR: 0.77; 95% CI: 0.61, 0.97) and not associated with liver cancer (RR: 0.96; 95% CI: 0.72, 1.28), while no meta-analysis has discussed the relationship between tofu and endometrial cancer or liver cancer. Tofu is rich in isoflavones, which have anti-cancer effects. Moreover, tofu contains a high calcium content, and the balance of calcium in the body is closely related to the occurrence and development of tumors [102]. Huang et al. [103] found that soy foods such as tofu may increase the number of beneficial bacteria (such as bifidobacteria and lactobacilli) in the gut, which may be associated with a reduced risk of gastric cancer. There are few meta-analyses on the relationship between soymilk and cancer risk, primarily as part of subgroup analyses of soy products. Previous meta-analyses found an inverse association between soymilk and gastric cancer [21,23], which is generally consistent with our study. Only two gastrointestinal cancer studies were included in our meta-analysis (one gastric cancer study and one colorectal cancer study), so the results should be interpreted cautiously. To date, there has been no meta-analysis of the relationship between soymilk and gynecological cancer risk, and as far as our findings are concerned, high soymilk consumption was not associated with breast and endometrial cancer.

As for the relationship between other soy products and total cancer risk. Our findings suggest that high consumption of soy paste is not associated with gastrointestinal cancer, particularly gastric cancer, which is consistent with previous meta-analyses [21,97]. In addition, high soy paste consumption can reduce the risk of breast cancer by 18%, in line with the results of Qin et al. [30]. On the other hand, very few studies discuss the association between soy paste and upper aerodigestive tract cancer. Soy paste was not associated with upper aerodigestive tract cancer, but the sample size was small (n = 2). Some researchers have found that miso soup intake can increase the risk of stomach cancer [21,97], and our study had no such association (RR: 1.12; 95% CI: 0.78, 1.61). Lu et al. [25] found that the intake of miso soup was not associated with gastrointestinal cancer but did not differentiate between gastric and colorectal cancer. A previous meta-analysis also found no association between miso soup and breast cancer, consistent with our findings [27]. Regarding natto, there is no meta-analysis on its association with cancer risk. Our meta-analysis included two studies on the relationship between natto and breast cancer, which were not associated with each other. Soy products can be categorized into fermented and non-fermented soy products according to different production processes. Our study found that high consumption of non-fermented soy products can reduce the risk of gastric cancer by 35% (RR: 0.96; 95% CI: 0.79, 1.17), while there was no association between fermented soy products and gastric cancer risk (RR: 0.65; 95% CI: 0.52, 0.80). Similar conclusions were reached by Weng et al. [97]. However, some studies have found that fermented soy products increase the risk of gastric cancer [21,104]. It is worth noting that this study only included studies that reported total fermented or non-fermented soy products rather than pooling various soy products together. This may lead to differences with the results of other meta-analyses.

Heterogeneity was presented in the study of total soy products, soymilk, soy paste, fermented soy products, and non-fermented soy products. A random effects model was used to increase the credibility of the findings. Subgroup analyses and meta-regression were performed to explore the sources of heterogeneity. When the studies were stratified by cancer type, heterogeneity in studies of non-fermented soy products and soy paste tended to disappear. Similarly, when the studies were stratified by the type of study design, the heterogeneity of total soy products and soymilk tended to disappear, and the heterogeneity of soymilk may also come from geographic location. Heterogeneity in fermented soy products may be due to geographic location and gender. Finally, a sensitivity analysis was performed, and the results were stable.

This meta-analysis has some of the following advantages. First, this is a comprehensive meta-analysis that analyzes the relationship between almost all common soy products and the risk of various types of cancer and makes an overall estimate of total cancer risk. Second, many recent studies with a large number of participants were included. Third, subgroup and meta-regression analyses were performed to examine the heterogeneity factors, and dose–response meta-analysis was applied to assess quantitatively the association between soy products and cancer risk. This meta-analysis likewise has some limitations. This study included case–control and cohort studies, with a majority of case–control studies. Therefore, the influence of methodological bias, such as recall bias, should be considered. For the relationship between total soy products, tofu, and soymilk and total cancer risk, there is a difference between the combined results of case–control studies and the combined results of the cohort studies, which requires careful interpretation. Moreover, the range and the cut-off values for soy product consumption varied among the studies, which may have biased the association between soy products and total cancer risk. To address this issue, dose–response meta-analysis was conducted. Furthermore, some evidence of bias was detected in the Begg and Egger tests for the analysis of total soy products, tofu, and total cancer risk. To counter this, the trim-and-fill method was performed and showed that publication bias did not affect the results. Lastly, although all studies adjusted for age, there were differences between studies for other confounders, such as total energy intake, which can impact the results of epidemiologic analysis [105].

## 5. Conclusions

In conclusion, our analysis suggests that high soy product consumption, especially tofu and soymilk, is associated with reduced cancer risk, particularly gastrointestinal and gynecological cancers. Increasing the daily intake of 54 g of total soy products reduces cancer risk by 11%, 61 g of tofu reduces cancer risk by 12%, and 23 g of soymilk reduces cancer risk by 28%. Evidence for an association between high consumption of other soy products (soy paste, miso soup, natto) and cancer risk remains insufficient. Finally, more well-designed prospective cohort studies on soy products and cancer should be conducted to confirm these findings.

## Figures and Tables

**Table 1:** Main characteristics of cohort/case–control studies in the meta-analysis.

First Author, Year	Country (Study Name)	Study Design (Study Period)	Age (Years)	Cases/ Sample	Exposure Category (Lowest vs. Highest)	Cancer Type
Chyou, 1995 [76]	USA	Cohort (1965–1993)	45–68	92/7994	Miso soup, tofu <1 times/week (ref) ≥5 times/week	Upper aerodigestive tract
Galanis, 1998 [34]	USA	Cohort (1975–1994)	46.4 ± 16.6	108/11,907	Miso soup None (ref), 1 or more times/week	Gastric
Nomura, 2004 [77]	USA	Cohort (1971–1995)	NA	304/5826	Tofu 0 g/week (ref), >240 g/week	Prostate
Oba, 2007 [40]	Japan (Takayama Study)	Cohort (1993–2000)	>35	210/30,221	Soy product 49.2 g/day (ref), 141.1 g/day (M) 46.3 g/day (ref), 128.0 g/day (F)	Colon
Akhter, 2008 [35]	Japan (Japan Public Health Center-Based Prospective Study)	Cohort (1995–2004)	45–74	886/83,063	Soy food 35.4 g/day (ref), 169.9 g/day (M) 35.6 g/day (ref), 170.3 g/day (F) Miso soup 147.5 mL/day (ref) 313.7 mL/day (M) 125.6 mL/day (ref) 261.3 mL/day (F)	Colorectal
Seow, 2009 [78]	Singapore (Singapore Chinese Health Study)	Cohort (1993–2005)	45–74	298/34,028	Soybean drink 30.7 g/day (ref), 197.7 g/day	Lung
Wang, 2009 [79]	USA (The Women’s Health Study)	Cohort (1992–2007)	≥45	3196/37,938	Tofu <1 serving/month (ref) ≥2 servings/week	Total
Yang, 2009 [80]	China (Shanghai Women’s Health Study)	Cohort (1997–2005)	51.6 ± 9	321/68,412	Soy foods ≤12.8 g/day (ref), >21 g/day	Colorectal
Hara, 2012 [36]	Japan (Japan Public Health Center-Based Prospective Study)	Cohort (1995–2006)	45–74	1249/84,881	Miso soup 63 mL/day (ref), 449 mL/day (M) 47 mL/day (ref), 384 mL/day (F) Soy food 33.4 g/day (ref), 140.6 g/day (M) 33.6 g/day (ref), 141 g/day (F)	Gastric
Ollberding, 2012 [81]	USA (Multiethnic Postmenopausal Women’s Cohort Study)	Cohort (1993–2007)	61.6 ± 7.7	489/46,027	Tofu 0–0.21 g/1000 kcal/day (ref) ≥7.56 g/1000 kcal/day	Endometrial
Ko, 2013 [47]	Korea (Korean Hereditary Breast Cancer Study)	Cohort (2007–2011)	≥20	2002/2271	Soybean products 0–1 times/week (ref) 4–5 times/week	Breast
Wada, 2015 [19]	Japan (Takayama Study)	Cohort (1992–2008)	>35	678/30,792	Soy foods 38.4 g/day (ref), 176.3 g/day (M) 43.5 g/day (ref), 168.7 g/day (F) Fermented soy foods 6.6 g/day (ref), 37.3 g/day (M) 7.5 g/day (ref), 34.0 g/day (F) Non-fermented 27.7 g/day (ref), 147.8 g/day (M) 32.0 g/day (ref), 140.8 g/day (F)	Stomach
Paul, 2019 [82]	Singapore (Singapore Chinese Health Study)	Cohort (1993–2013)	45–74	312/30,744	Soy food 31.29 g/1000 kcal/day 115.86 g/1000 kcal/day	Cervical
Abe, 2020 [20]	Japan (Japan Public Health Center-Based Prospective Study)	Cohort (1995–2013)	40–69	534/75,089	Miso, natto, tofu, fermented Non-fermented, total soy food Quartile 1 (ref), quartile 4 Soymilk Non-consumer (ref), consumer	Liver
Shirabe, 2021 [83]	Japan (Japan Public Health Center-Based Prospective Study)	Cohort (1995–2013)	45–74	825/47,614	Total soy foods 31.8 g/day (ref), 137 g/day Fermented soy foods 7.3 g/day (ref), 53.2 g/day Miso 2.9 g/day (ref), 29.1 g/day Natto 0.01 g/day (ref), 32.7 g/day Non-fermented soy foods 13.5 g/day (ref), 98.5 g/day Tofu 10.3 g/day (ref), 74.1 g/day Soy milk No (ref), Yes	Breast
Nozue, 2021 [37]	Japan (Japan Public Health Center-based Prospective Study)	Cohort (1995–2012)	40–69	9972/79,648	Total soy product 33.6 g/day (ref), 140.9 g/day (W) 33.7 g/day (ref), 130.9 g/day (M) Fermented soy products 8.6 g/day (ref), 55.1 g/day (W) 9.5 g/day (ref), 60 g/day (M) Non-fermented soy products 13.5 g/day (ref), 99.5 g/day (W) 12.1 g/day (ref), 97 g/day (M) Miso 3.8 g/day (ref), 30.5 g/day (W) 5.1 g/day (ref), 35.8 g/day (M) Natto 0 g/day (ref), 32.4 g/day (W) 0 g/day (ref), 32.1 g/day (M)	Total
Shin, 2023 [17]	Korea (the Health Examinees study)	Cohort (2004–2013)	40–69	767/109,161	Soybean paste, tofu Almost never (ref) ≥2 times/week	Gastric
Lee, 1995 [12]	Korea	Case–control (1990–1991)	>25	213/425	Tofu None or 4–5 times/year (ref) ≥2–3 times/week	Stomach
Horn-Ross, 2001 [56]	USA (Multiethnic Bay Area Breast Cancer Study)	Case–control (1995–1998)	35–79	1314/2917	Tofu, miso soup Non-consumers (ref) ≥1 times/month Soy milk Non-consumers (ref), consumers	Breast
Seow, 2002 [45]	Singapore (Singapore Chinese Women’s health study)	Case–control (1996–1998)	20–89	303/1064	Soy foods <2.2 servings/week (ref) ≥5.4 servings/week	Lung
Wu, 2002 [57]	USA	Case–control (1995–1998)	25–74	494/1086	Tofu Less than monthly (ref) >4 times/week	Breast
Hirose, 2003 [44]	Japan (Aichi Cancer Center-Based Women’s Health Study)	Case–control (1988–2000)	>30	2382/21,377	Soybean curd <1–3 times/month ≥5 times/week Miso soup Almost never (ref), 2 times/day	Breast
Lee, 2003 [13]	Korea	Case–control (1999)	>18	69/268	Soybean curd <1 times/week (ref) ≥1 times/month	Gastric
Jian, 2004 [58]	China	Case–control (2001–2002)	>45	130/404	Fermented soy products 0 g/day (ref), >4 g/day	Prostate
Hung, 2004 [42]	China	Case–control (1996–2002)	Case (41–93) Control (41–89)	522/1428	Fermented bean product <1 (ref), ≥1 times/week	Esophageal
Machida-montani, 2004 [59]	Japan	Case–control (1998–2002)	20–74	122/357	Miso soup <3 (ref), ≥4 cups/day	Gastric
Sonoda, 2004 [60]	Japan	Case–control (1996–2002)	59–73	140/280	Tofu ≤19.7 g/day (ref), ≥96.4 g/day All soy products ≤77 g/day (ref), ≥187.2 g/day Natto ≤5.7 g/day (ref), ≥40 g/d	Prostate
Xu, 2004 [61]	China (Shanghai Women’s Population-Based Case–control Study)	Case–control (1997–2001)	30–69	832/1678	Soy milk Never (ref), >1.9 g/day Tofu ≤0.8 (ref), >3.5 g/day Soya products (no tofu) ≤1.8 (ref), >8.8 g/day	Endometrial
Nan, 2005 [5]	Korea	Case–control (1997–2003)	Case 60 ± 11 Control 59 ± 10	421/1053	Soybean paste Low (ref), high	Gastric
Hirose, 2005 [14]	Japan (Aichi Cancer Center-Based Women’s Health Study)	Case–control (2001–2002)	>30	167/1021	Soybean products, tofu, Miso, natto Tertile 1 (ref), Tertile 3	Breast
Sharp, 2005 [62]	Japan (A-bomb Survivors Cohort-Based, Case–control Study)	Case–control (1965–1988)	NA	102/339	Miso soup, tofu Never or ≤1/week (ref) ≥5 times/week	Hepatocellular
Shannon, 2005 [63]	China (Shanghai Women’s Study)	Case–control (1995–2000)	>35	378/1448	Total soy food ≤2.6 servings/week (ref) ≥1.1 servings/day Unfermented soy food ≤2.3 servings/week (ref) ≥1 servings/day	Breast
Heald, 2007 [64]	Scottish (Prostate Cancer And Diet Study)	Case–control (1998–2001)	50–74	433/916	Soy food consumption No (ref), Yes	Prostate
Kim, 2008 [65]	Korea	Case–control (2004–2006)	Case 46.1 ± 8.5 Control 46 ± 8.6	362/724	Tofu <7.73 g/day (ref), ≥49.5 g/day	Breast
Li, 2008 [66]	China (Changchun Mass Screening-Based Case–control study)	Case–control (1998–2000)	>50	28/308	Soybean food (tofu and foymilk) ≤2 times/day (ref) ≥1 times/day	Prostate
Matsuo, 2008 [16]	Japan (Aichi Cancer Center Hospital-Based Case–control Study)	Case–control (2001–2005)	18–79	353/2110	Soybean products 19.8 (ref), 81.8 g/day Miso soup ≤3–4 times/week (ref) twice a day Tofu ≤1–3 times/month (ref) ≥3–4 times/week Natto ≤1–3 times/month (ref) ≥ once a day	Lung
Bandera, 2009 [67]	USA (Estrogen, Diet, Genetics and Endometrial Cancer Study)	Case–control (2001–2003)	>21	408/797	Tofu, soy milk, miso Never (ref), Ever	Endometrial
Liu, 2009 [68]	China	Case–control (1997–2005)	2–20	195/683	Bean curd foods Rare or occasional (ref), frequent	Leukemia
Hosono, 2010 [46]	Japan	Case–control (2001–2005)	NA	405/2430	Tofu None (ref), >5 times/week	Cervical
Budhathoki, 2011 [41]	Japan (The Fukuoka Colorectal Cancer Study)	Case–control (2000–2003)	Case 60.5 ± 9.1 Control 58.9 ± 10.7	816/1631	Soy foods 5.4 g/day (ref), 26.8 g/day	Colorectal
Chihara, 2012 [38]	Japan	Case–control (2001–2005)	18–80	295/1765	Miso soup, tofu, natto <1 times/day (ref), ≥1 times/day	Non-Hodgkin lymphoma
Wang, 2012 [69]	China (Northwest China’s Hospital-Based Case–control Study)	Case–control (2009–2011)	NA	220/440	Soy food Never (ref), ≥3 times/week	Multiple myeloma
Wang, 2012 [70]	China (XiAn’s Population- Based Case–control Study)	Case–control (2008–2010)	30–79	257/771	Soya products Tertile 1 (ref), Tertile 3	Gastric
Zaineddin, 2012 [71]	Germany (German Case–control Study)	Case–control (2005–2006)	50–74	3157/9211	Soy milk, tofu, paste No consumption (ref) High consumption	Breast
Lee, 2014 [72]	China (Guangdong Hospital-Based 1:1 Case–control Study)	Case–control (2006–2008)	Average 75	500/1000	Total soy foods ≤61.4 g/day (ref), >119 g/day Soy milk ≤12.9 mL/day (ref), >38.6 mL/day Tofu ≤8.6 g/day (ref), >20 g/day	Ovarian
Tang, 2015 [73]	China (Xinjiang Hospital-Based Case–control Study)	Case–control (2008–2009)	Average 61	359/539	Total soya foods <26 g/day (ref), >97 g/day Soya milk <2 mL/day (ref), >60 mL/day	Esophageal
Shin, 2015 [15]	Korea	Case–control (2010–2013)	NA	962/3727	Soy products <40.34 g/day (ref) ≥105.03 g/day (M) <42.77 g/day (ref) ≥113.66 g/day (F) Tofu <17.19 g/day (ref) ≥52.86 g/day (M) <18.73 g/day (ref) ≥54.91 g/day (F) Soymilk 0 g/day (ref), ≥21.35 g/day (M) 0 g/day (ref), ≥19.1 g/day (F) Fermented soy paste <1.95 g/day (ref) ≥8.32 g/day (M) <2.08 g/day (ref) ≥8.7 g/day (F)	Colorectal
Yang, 2017 [18]	Korea (National Cancer Center Gastric Cancer Research)	Case–control (2011–2014)	Case 53.9 ± 9.19 Control 53.8 ± 9.05	377/1131	Total soy products ≤48.39 g/day (ref), >86.2 g/day Fermented soy foods ≤2.29 g/day (ref), >5.78 g/day Non-fermented soy foods ≤42.95 g/day (ref), >85.54 g/day Tofu ≤20.47 g/day (ref), >40.05 g/day Soymilk ≤4.24 × 10^−9^ g/day (ref), ≥3.55 g/day	Gastric
Barrett, 2019 [43]	China (NPC Genes, Environment, and EBV Study)	Case–control (2010–2013)	20–74	4806/9614	Fermented bean curds (adult) 0 g/day (ref), ≥0.66 g/day (M) 0 g/day (ref), >0.33 g/day (W) Bean paste (adult) 0 g/day (ref), ≥1.66 g/day Fermented bean curds (adolescent) 0 g/day (ref), ≥0.66 g/day (M) 0 g/day (ref), >0.54 g/day (W) Bean paste (adolescent) 0 g/day (ref), >2.5 g/day	Nasopharyngeal carcinoma
Cao, 2022 [74]	China (Chinese Wuxi Exposure and Breast Cancer Study)	Case–control (2013–2014)	>18	818/1753	Soy foods 0–3.3 g/day (ref), ≥57.1 g/day	Breast
Teng, 2023 [39]	China	Case–control (2018–2019)	25–80	113/405	Soybean products 0–10 g/day (ref) 41.8–181.7 g/day	Bladder
Kwak, 2023 [75]	Korea	Case–control (2002–2006)	20–70	82/164	Soy products Tertile 1 (ref), Tertile 3	Gastric

M: male; F: female; NA: not available; ref: reference.

**Table 2:** Pooled RRs of cancer risk for the highest versus lowest categories of total soy product, tofu and soy milk consumption.

Characteristic	Studies (n)	RR (95% CI)	Heterogeneity	*p*-Difference
Total soy foods				
All studies	28	0.69 (0.6, 0.8)	*I*^2^ = 82.7%, *p* < 0.001	
Study design				
Case–control study	18	0.56 (0.46, 0.69)	*I*^2^ = 69.8%, *p* < 0.001	*p* = 0.004
Cohort study	10	0.90 (0.80, 1.01)	*I*^2^ = 66.8%, *p* = 0.001	
Sex				
Male	12	0.86 (0.74, 1.00)	*I*^2^ = 59.0%, *p* = 0.005	*p* = 0.454
Female	20	0.76 (0.65, 0.89)	*I*^2^ = 78.3%, *p* < 0.001	
Cancer type				
Gastrointestinal cancer	10	0.74 (0.61, 0.89)	*I*^2^ = 68.7%, *p* = 0.001	
Stomach	5	0.63 (0.41, 0.97)	*I*^2^ = 81.5%, *p* < 0.001	
Colorectal	5	0.77 (0.65, 0.91)	*I*^2^ = 43.1%, *p* = 0.134	
Gynecological cancer	8	0.71 (0.54, 0.92)	*I*^2^ = 83.3%, *p* < 0.001	*p* = 0.902
Breast	5	0.79 (0.60, 1.03)	*I*^2^ = 73.9%, *p* = 0.004	
Ovarian	1	0.29 (0.20, 0.42)		
Cervical	1	0.80 (0.61, 1.05)		
Endometrial	1	0.84 (0.59, 1.20)		
Prostate cancer	3	0.47 (0.31, 0.71)	*I*^2^ = 0%, *p* = 0.566	*p* = 0.163
Lung cancer	2	0.67 (0.52, 0.86)	*I*^2^ = 0%, *p* = 0.564	*p* = 0.608
Bladder cancer	1	0.75 (0.40, 1.41)		*p* = 0.983
Upper aerodigestive tract cancer	1	0.33 (0.22, 0.49)		*p* = 0.062
Multiple myeloma	1	0.10 (0.01, 0.97)		*p* = 0.257
Liver cancer	1	1.10 (0.82, 1.47)		*p* = 0.296
Geographic location				
Japan	10	0.89 (0.77, 1.02)	*I*^2^ = 65.7%, *p* = 0.002	
China	11	0.48 (0.34, 0.69)	*I*^2^ = 80.8%, *p* < 0.001	*p* = 0.022
Korea	4	0.75 (0.61, 0.92)	*I*^2^ = 40.0%, *p* = 0.172	*p* = 0.241
Singapore	2	0.73 (0.57, 0.94)	*I*^2^ = 14.1%, *p* = 0.281	*p* = 0.285
USA	1	0.73 (0.51, 1.04)		*p* = 0.488
Europe	1	0.52 (0.60, 0.80)		*p* = 0.160
Tofu				
All studies	26	0.78 (0.70, 0.86)	*I*^2^ = 47.9%, *p* = 0.004	
Study design				
Case–control study	19	0.72 (0.63, 0.83)	*I*^2^ = 58.9%, *p* = 0.001	*p* = 0.186
Cohort study	7	0.89 (0.78, 1.01)	*I*^2^ = 0%, *p* = 0.901	
Sex				
Male	7	0.83 (0.71, 0.98)	*I*^2^ = 20.3%, *p* = 0.268	*p* = 0974
Female	19	0.82 (0.74, 0.91)	*I*^2^ = 34.7%, *p* = 0.069	
Cancer type				
Gastrointestinal cancer	5	0.67 (0.47, 0.96)	*I*^2^ = 76.0%, *p* = 0.002	
Stomach	4	0.56 (0.34, 0.93)	*I*^2^ = 79.7%, *p* = 0.002	
Colorectal	1	0.95 (0.73, 1.24)		
Gynecological cancer	12	0.76 (0.66, 0.87)	*I*^2^ = 48.7%, *p* = 0.029	*p* = 0898
Breast	7	0.79 (0.66, 0.94)	*I*^2^ = 61.6%, *p* = 0.016	
Endometrial	3	0.77 (0.61, 0.97)	*I*^2^ = 0%, *p* = 0.785	
Cervical	1	0.62 (0.34, 1.14)		
Ovarian	1	0.57 (0.40, 0.81)		
Prostate cancer	2	0.70 (0.43, 1.15)	*I*^2^ = 26.0%, *p* = 0.245	*p* = 0.917
Liver cancer	2	0.96 (0.72, 1.28)	*I*^2^ = 0%, *p* = 0.931	*p* = 0.465
Lung cancer	1	0.96 (0.70, 1.32)		*p* = 0.542
Non-Hodgkin lymphoma	1	1.35 (0.75, 2.44)		*p* = 0.341
Upper aerodigestive tract cancer	1	0.49 (0.07, 3.48)		*p* = 0.874
Leukemia	1	0.55 (0.34, 0.89)		*p* = 0.898
Geographic location				
Japan	9	0.87 (0.76, 1.00)	*I*^2^ = 20.0%, *p* = 0.265	
USA	7	0.82 (0.71, 0.95)	*I*^2^ = 0%, *p* = 0.863	*p* = 0.546
Korea	6	0.58 (0.40, 0.85)	*I*^2^ = 80.7%, *p* < 0.001	*p* = 0.169
China	3	0.63 (0.50, 0.80)	*I*^2^ = 1.4%, *p* = 0.363	*p* = 0.049
Europe	1	0.89 (0.74, 1.08)		*p* = 0.876
Soy milk				
All studies	11	0.75 (0.60, 0.93)	*I*^2^ = 80.6%, *p* < 0.001	
Study design				
Case–control study	8	0.65 (0.52, 0.80)	*I*^2^ = 72.4%, *p* = 0.001	*p* = 0.031
Cohort study	3	1.10 (0.76, 1.58)	*I*^2^ = 72.9%, *p* = 0.025	
Sex				
Male	3	0.72 (0.37, 1.41)	*I*^2^ = 90.4%, *p* < 0.001	*p* = 0.699
Female	10	0.81 (0.65, 1.00)	*I*^2^ = 71.8%, *p* < 0.001	
Cancer type				
Gastrointestinal cancer	2	0.58 (0.47, 0.72)	*I*^2^ = 0%, *p* = 0.759	
Stomach	1	0.61 (0.43, 0.87)		
Colorectal	1	0.57 (0.44, 0.73)		
Gynecological cancer	6	0.79 (0.58, 1.06)	*I*^2^ = 82.2%, *p* < 0.001	*p* = 0.359
Breast	3	0.91 (0.60, 1.38)	*I*^2^ = 80.9%, *p* = 0.005	
Endometrial	2	0.86 (0.69, 1.06)	*I*^2^ = 0%, *p* = 0.964	
Ovarian	1	0.43 (0.31, 0.6)		
Upper aerodigestive tract cancer	1	0.48 (0.31, 0.74)		*p* = 0.569
Lung cancer	1	0.75 (0.53, 1.06)		*p* = 0.150
Liver cancer	1	1.31 (0.95, 1.80)		*p* = 0.433
Geographic location				
Japan	2	1.32 (1.05, 1.66)	*I*^2^ = 0%, *p* = 0.922	
China	3	0.57 (0.35, 0.93)	*I*^2^ = 84.7%, *p* = 0.001	*p* = 0.068
USA	2	0.69 (0.47, 1.02)	*I*^2^ = 41.8%, *p* = 0.190	*p* = 0.074
Korea	2	0.58 (0.47, 0.72)	*I*^2^ = 0%, *p* = 0.759	*p* = 0.035
Singapore	1	0.75 (0.53, 1.06)		*p* = 0.225
Europe	1	0.94 (0.75, 1.18)		*p* = 0.284

**Table 3:** Pooled RRs of cancer risk for the highest versus lowest categories of soy paste, miso soup, natto, fermented soy foods and unfermented soy foods consumption.

Characteristic	Studies (n)	RR (95% CI)	Heterogeneity	*p*-Difference
Soy paste				
All studies	12	0.99 (0.87, 1.13)	*I*^2^ = 75.2%, *p* < 0.001	
Study design				
Case–control study	8	1.06 (0.85, 1.33)	*I*^2^ = 82.1%, *p* < 0.001	*p* = 0.271
Cohort study	4	0.95 (0.89, 1.01)	*I*^2^ = 0%, *p* = 0.401	
Sex				
Male	7	0.93 (0.71, 1.22)	*I*^2^ = 78.1%, *p* < 0.001	*p* = 0.884
Female	11	0.92 (0.82, 1.02)	*I*^2^ = 18.8%, *p* = 0.264	
Cancer type				
Gastrointestinal cancer	4	1.23 (0.88, 1.74)	*I*^2^ = 84.7%, *p* < 0.001	
Stomach	3	1.13 (0.74, 1.73)	*I*^2^ = 80.6%, *p* = 0.001	
Colorectal	1	1.60 (1.25, 2.05)		
Gynecological cancer	5	0.87 (0.72, 1.05)	*I*^2^ = 32.2%, *p* = 0.207	*p* = 0.117
Breast	4	0.82 (0.70, 0.96)	*I*^2^ = 0%, *p* = 0.520	
Endometrial	1	1.31 (0.83, 2.07)		
Upper aerodigestive tract cancer	1	0.90 (0.79, 1.02)		*p* = 0.441
Liver cancer	1	0.81 (0.60, 1.10)		*p* = 0.352
Geographic location				
Japan	4	0.88 (0.75, 1.03)	*I*^2^ = 39.4%, *p* = 0.175	
Korea	4	1.23 (0.88, 1.74)	*I*^2^ = 84.7%, *p* < 0.001	*p* = 0.093
China	2	0.90 (0.80, 1.02)	*I*^2^ = 0%, *p* = 0.900	*p* = 0.997
USA	1	1.31 (0.83, 2.07)		*p* = 0.236
Europe	1	0.79 (0.61, 1.02)		*p* = 0.593
Miso soup				
All studies	10	0.99 (0.87, 1.12)	*I*^2^ = 15.3%, *p* = 0.302	
Study design				
Case–control study	6	0.98 (0.76, 1.27)	*I*^2^ = 39.2%, *p* = 0.144	*p* = 0.780
Cohort study	4	0.97 (0.84, 1.11)	*I*^2^ = 0%, *p* = 0.514	
Sex				
Male	5	1.01 (0.77, 1.32)	*I*^2^ = 50.4%, *p* = 0.089	*p* = 0.682
Female	7	0.97 (0.84, 1.12)	*I*^2^ = 0%, *p* = 0.508	
Cancer type				
Gastrointestinal cancer	4	1.01 (0.83, 1.22)	*I*^2^ = 34.1%, *p* = 0.207	
Stomach	3	1.12 (0.78, 1.61)	*I*^2^ = 55.5%, *p* = 0.106	
Colorectal	1	0.96 (0.78, 1.19)		
Breast cancer	2	1.05 (0.86, 1.28)	*I*^2^ = 0%, *p* = 0.681	*p* = 0.648
Liver cancer	1	0.5 (0.15, 1.66)		*p* = 0.442
Lung cancer	1	0.53 (0.28, 1.01)		*p* = 0.234
Upper aerodigestive tract cancer	1	1.63 (0.51, 5.21)		*p* = 0.539
Non-Hodgkin lymphoma	1	1.01 (0.60, 1.70)		*p* = 0.933
Geographic location				
Japan	7	0.94 (0.80, 1.10)	*I*^2^ = 24.7%, *p* = 0.240	*p* = 0.188
USA	3	1.15 (0.91, 1.47)	*I*^2^ = 0%, *p* = 0.791	
Natto				
All studies	7	0.96 (0.82, 1.11)	*I*^2^ = 33.8%, *p* = 0.170	
Study design				
Case–control study	4	0.74 (0.58, 0.95)	I2 = 0%, *p* = 0.592	*p* = 0.045
Cohort study	3	1.05 (0.98, 1.13)	I2 = 0%, *p* = 0.952	
Sex				
Male	5	0.96 (0.74, 1.26)	*I*^2^ = 57.6%, *p* = 0.051	*p* = 0.402
Female	6	0.99 (0.90, 1.10)	*I*^2^ = 0%, *p* = 0.680	
Cancer type				
Breast cancer	2	0.97 (0.73, 1.29)	*I*^2^ = 0%, *p* = 0.336	
Prostate cancer	1	0.25 (0.05, 1.24)		*p* = 0.350
Lung cancer	1	0.74 (0.51, 1.07)		*p* = 0.453
Non-Hodgkin lymphoma	1	0.76 (0.46, 1.24)		*p* = 0.550
Liver caner	1	1.10 (0.80, 1.51)		*p* = 0.67
Fermented soy foods				
All studies	7	1.18 (0.95, 1.47)	*I*^2^ = 77.3%, *p* < 0.001	
Study design				
Case-control study	3	1.74 (0.96, 3.15)	*I*^2^ = 81.8%, *p* = 0.004	*p* = 0.056
Cohort study	4	0.99 (0.93, 1.06)	*I*^2^ = 0%, *p* = 0.805	
Sex				
Male	5	1.03 (0.91, 1.17)	*I*^2^ = 19.3%, *p* = 0.292	*p* = 0.573
Female	5	0.98 (0.89, 1.09)	*I*^2^ = 0%, *p* = 0.460	
Cancer type				
Stomach cancer	2	0.96 (0.79, 1.17)	*I*^2^ = 0%, *p* = 0.393	
Breast cancer	1	0.94 (0.67, 1.32)		*p* = 0.935
Prostate cancer	1	2.02 (1.08, 3.78)		*p* = 0.270
Liver cancer	1	1.08 (0.78, 1.50)		*p* = 0.656
Upper aerodigestive tract cancer	1	2.55 (1.71, 3.80)		*p* = 0.146
Geographic location				
Japan	4	0.99 (0.93, 1.06)	*I*^2^ = 0%, *p* = 0.805	
China	2	2.38 (1.70, 3.34)	*I*^2^ = 0%, *p* = 0.539	*p* = 0.008
Korea	1	1.08 (0.77, 1.51)		*p* = 0.698
Unfermented soy foods				
All studies	6	0.95 (0.77, 1.18)	*I*^2^ = 75.8%, *p* = 0.001	
Study design				
Case–control study	2	0.86 (0.44, 1.67)	*I*^2^ = 80.7%, *p* = 0.023	*p* = 0.624
Cohort study	4	1.00 (0.80, 1.25	*I*^2^ = 76.3%, *p* = 0.005	
Sex				
Male	4	0.81 (0.60, 1.10)	*I*^2^ = 82.6%, *p* = 0.001	*p* = 0.239
Female	6	1.08 (0.93, 1.25)	*I*^2^ = 24.6%, *p* = 0.249	
Cancer type				
Stomach cancer	2	0.65 (0.52, 0.80)	*I*^2^ = 0%, *p* = 0.789	
Breast cancer	2	1.17 (0.91, 1.51)	*I*^2^ = 0%, *p* = 0.832	*p* = 0.073
Liver cancer	1	1.21 (0.92, 1.60)		*p* = 0.178
Geographic location				
Japan	4	1.00 (0.80, 1.25)	*I*^2^ = 76.3%, *p* = 0.005	
China	1	1.22 (0.78, 1.91)		*p* = 0.613
Korea	1	0.62 (0.43, 0.90)		*p* = 0.253

## Data Availability

The data presented in this study are available upon request from the corresponding author. The data are not publicly available due to privacy.

## Supplementary Materials

The following supporting information can be downloaded at: https://www.mdpi.com/article/10.3390/nu16070986/s1. Table S1: PRISMA 2020 Checklist for this systematic review and meta-analysis; Table S2: Covariates and Nos score of included studies for this systematic review and meta-analysis; Table S3: Quality assessment of included studies for this systematic review and meta-analysis; Table S4: Exposure categories and ORs of included studies for this systematic review and meta-analysis.
