# Supplementary material for: Soy Product Consumption and the Risk of Cancer: A Systematic Review and Meta-Analysis of Observational Studies
Source: Nutrients. 2024 Mar 28;16(7):986. doi: 10.3390/nu16070986 (PMC11013307; doi:10.3390/nu16070986)
Supplement: Supplementary file 1 [file nutrients-16-00986-s001.zip › Table S2.pdf]

Supplementary Table S2. Covariates and Nos score of included studies for this systematic review and meta-analysis.

First author, Year	Adjustments	Nos score
Chyou,1995	Age, Alcohol intake,Smoking	7
Galanis,1998	Age, Alcohol intake, Birth place, Education, Sex, Smoking	9
Nomura,2004	Age, Alcohol intake, Arm muscle area, BMI, Smoking, Total calories	8
Oba,2007	Age, Alcohol intake, BMI, Coffee intake, Height, Physical exercise, Smoking, and Use of hormone replacement therapy	9
Akhter,2008	Age, Alcohol intake, BMI, Dairy products, History of diabetes mellitus, Intake of vitamin D, meat, vegetable, friut, and fish, Menopausal status and Current use of femal hormones, Physical activity, Public health center area, Smoking	8
Seow,2009	Age, BMI, Education, Smoking, Year of interview, Dialect group, Total vegetable intake, Total fruit/juice intake, h-cryptoxanthin, Total isothiocyanates	8
Wang,2009	Age, Alcohol intake, BMI, Family history of colorectal cancer, ovary cancer, or breast cancer, Hormone replacement therapy use, Intake of fruit and vegetables, fiber, folate, saturated fat Multivitamin use, Physical activity, Postmenopausal status, Race, Randomized treatment, Total energy intake, Smoking	5
Yang,2009	Age, BMI, Education, Family history of colorectal cancer, Household income, Physical activity, Menopausal status, Total calorie intake and average intakes of fruit, vegetables, red meat, nonsoy calcium, nonsoy fiber, and nonsoy folic acid and was stratified by birth year	8
Hara,2012	Age and Public center area, BMI, Smoking, Ethanol intake, Family history of gastric cancer, Fruit intake, fish intake, Salt intake, Total energy intake, Vegetable intake	8
Ollberding, 2012	Age, Age at cohort entry, Age at menarche, Age at menopause, BMI, Current unopposed estrogen use, Diabetes, Duration and type of hormone therapy use, Duration of oral contraceptive, Parity, Race, Smoking, Total calories	6
Ko,2013	Age at menarche, Alcohol intake, Calorie intake, Education, Parity, Physical exercise, Smoking,	4
Wada,2015	Age, Alcohol intake, BMI, Education, Menopausal status, Physical activity, Smoking, Salt intake	9
Paul,2019	Age, Dialect group, Duration of oral contraceptive use, Education, History of Pap-based test, Menopausal status, and Daily total calorie intake, Parity, Smoking, Year of interview	8
Abe,2020	Age, Area, Smoking, Alcohol intkae, Age at menarche, Age at first birth, Number of deliveries, Menopausal age, Hormone use, and Breast feeding, BMI, Height, Energy intake, History of diabetes, Family history, Physical activity, Received mammography	6

Shirabe,2021	Age, Area,Smoking, Alcohol intkae, Age at menarche, Age at first birth, Number of deliveries, Menopausal age, Hormone use, and Breast feeding, BMI, Height, Energy intake, History of diabetes, Family history, Physical activity, Received mammography	6
Nozue, 2021	Age, Alcohol intake, BMI, Intakes of total energy, vegertables, fruit, fish, green tea and coffee, Menopausal status, Metabolic equivalent task hours, Public health center area, Smoking	7
Shin, 2023	Age, Alcohol intake, BMI, Education, Regular exercise, Smoking, Sodium intake, The presence of a family history of gastric cancer, Total energy intake, vegetable intake	7
Lee,1995	Age, Area, Education, Economic status, Other dietary factors,Sex	6
Horn-Ross, 2001	Age, Age at menarche, BMI, Education, Daily caloric intake, Ethnicity, Family history of breast cancer, Histroy of benign breast cancer, Parity, A composite variable including menopausal status,Hormone replacement therapy use	8
Seow,2002	Age, Fruit intake, First-degree relative with history of cancer, Place of birth, Smoking	8
Wu,2002	Age, Alcohol intake, Age at menarche, Asian-ethnicity, BMI, Birth place, Education, Family history of breast cancer, Intake of dark leafy greens, Menopausal status and use of menopausal, Pregnancy, Smoking, Physical activty	7
Hirose,2003	Age, Age at menarche, Famliy history, Virst year, Parity and Age at first full term pregnancy	7
Lee,2003	Age, Alcohol intake, Educaion, Family history of gastric cancer, H.pylori infecion, Sex, Smoking	8
Jian,2004	Age, Area, Caloric intake, BMI, Education, Fresh vegetables and fruits consumption, tea drinking, Famliy income, Martial status, MET, Prostate cancer in first-degree relatives	7
Hung,2004	Age, Alcohol intake, Education, Ethinicity, Source of hospital, Smoking and areca nut chewing	7
Machida-montani, 2004	Age, Family history of gastric cancer, H.pylori infection, JA menbership, Smoking, Salt intake , Total energy intake, Total vegetable intake, Total fruit intake	8
Sonoda,2004	Age, Energy intake, Smoking	6
Xu,2004	Age, BMI, Education, Energy intake, Family history of any cancer, Number of pregnancies, Menopausal status, Oral contraceptive use, Physical acticity, Total meat intake, Years of menstruation	7
Nan,2005	Age, Sex	5
Hirose,2005	Age, Alcohol intake, Age at menarche, Age at first full-term pregnancy, Age at menopause for postmenopausal women, BMI, Energy, Family history, Motives for consultation, Parity, Physical exercise, Smoking	7

Sharp,2005	Attained age, City, HBV and HCV, Liver irradiation level, Year of death, Sex	5
Shannon,2005	Age, Breast-feeding, Total energy intake	5
Heald,2007	Age, Carstairs Deprivation Index, Energy intake, Family history of prostate cancer, Smoking, Total energy intake	7
Kim,2008	Age, Breast feeding, Carbohydrate intake, Dietary factors, Drinking, Number of children, Multivitamin use	7
Li,2008	Age, Alcohol intake, BMI, Education, Food frequency, Smoking	7
Matsuo,2008	Age, Energy intake, Sex, Smoking	6
Bandera,2009	Age, Age at menarche, BMI,Education, Menopausal status and Age at menopause for postmenopausal women, OC use, Parity, Race, Total calories	7
Liu,2009	Age, Sex	7
Hosono,2010	Age, Alcohol intake, Any vitamin supplement usage and energy, Gravidity, OC usage, Smoking	7
Budhathoki 2011	Age, Area, Alcohol intake, BMI, Sex, Smoking, Intake of calcium and n-3 polyunsaturated fatty acids, Parental colorectal cancer, Type of job, Physical activity	8
Chihara,2012	Age, Alcohol intake, BMI at 20 years of age, Smoking, Total energy intake	6
Wang,2012	Age, Dietary risk factors, Education, Family history of cancer in first degree relatives, Sex	7
Wang,2012	Age, Alcohol intake, Education, Family history, Pickled food, Smoking, Total vegetable intake, Total fruit intake, Total energy intake, meat, and H pylori	6
Zaineddin, 2012	Alcohol intake,Age at menarche, BMI, Education, Breast feeding history, Energy intake, Fiber intake, Famliy history of breast cancer, History of benign breast disease, Number of pregnancies, Menopausal status, Total number of mammograms, Smoking, Phytoestrogen supplement use	5
Lee,2014	Age, Alcohol intake, BMI, Education, Famliy history of ovarian or breast cancer, Menopausal status, Oral contraceptive use, Physical acticity, Parity, Hormone replacement therapy, Total energy intake,Smoking	7
Tang,2015	Age, Aocohol intake, BMI, Education, Family history of cancer in first-degree relatives, Total energy intake, Sex, Smoking	7
Shin,2015	Age, Alcohol intake, Education, Physical exercise	5
Yang,2017	Age, Alcohol intake, Education, H pylori infection, Physical exercise, Smoking	7
Barrett,2019	Age, Area, Adolescent daily energy intake, Energy-adjusted intake of other foods and Childhood frequency of intake of total preserved foods, Current housing type, Current occupation, Education, First-degree family history of NPC, Smoking, Sex	7
Cao,2022	Age at diagnosis for cases or enrollment for controls, Area, Age at menarche,number of full-term birth, Age at fisrt full-term delivery, BMI,	5

	Breast feeding, Education, Famliy history of breast cancer, Hormonte replacement therapy, History of benign breast disease, Menopausal age, Oral contracepetives use, Physical activity, Smoking	
Teng,2023	Age, Alcohol intake, Education, Occupation, Sex, Smoking	8
Kwak,2023	Age, Alcohol intake, BMI, Education, Family history of gastric cancer, Hospital, Other food intake, Total energy intake, Sex, Smoking	6

[1] Nos, Newcastle-Ottwa Scale
