# Supplementary material for: Soy Product Consumption and the Risk of Cancer: A Systematic Review and Meta-Analysis of Observational Studies
Source: Nutrients. 2024 Mar 28;16(7):986. doi: 10.3390/nu16070986 (PMC11013307; doi:10.3390/nu16070986)
Supplement: Supplementary file 1 [file nutrients-16-00986-s001.zip › Table S3.pdf]

Supplementary Table S3. Quality assessment of included studies for this systematic review and meta-analysis.

Study	Selection of the study groups 0-4	Adjustment for Known confounding factors 0-2	Ascertainment of the outcome of interest 0-3	Total score
Chyou	4	1	2	7
Galanis	4	2	3	9
Nomura	3	2	3	8
Oba	4	2	3	9
Akhter	3	2	3	8
Seow	4	2	2	8
Wang	1	2	2	5
Yang	3	2	3	8
Hara	3	2	3	8
Ollberding	3	2	1	6
Ko	2	2	0	4
Wada	4	2	3	9
Paul	4	2	2	8
Abe	3	2	1	6
Shirabe	3	2	1	6
Nozue	3	2	2	7
Shin	3	2	2	7
Lee	2	2	2	6
Horn-Ross	4	2	2	8
Seow	3	2	3	8
Wu	3	2	2	7
Hirose	3	2	2	7
Lee	4	2	2	8
Jian	3	2	2	7
Hung	3	2	2	7
Machida-Montani	4	2	2	8
Sonoda	3	1	2	6
Xu	3	2	2	7
Nan	3	1	1	5
Hirose	3	2	2	7
Sharp	2	1	2	5
Shannon	2	1	2	5
Heald	3	2	2	7
Li	3	2	2	7
Kim	3	2	2	7
Matsuo	3	1	2	6

Bandera	3	2	2	7
Liu	4	1	2	7
Hosono	3	2	2	7
Budhathoki	4	2	2	8
Chihara	3	1	2	6
Wang	3	2	2	7
Wang	2	2	2	6
Zaineddin	1	2	2	5
Lee	3	2	2	7
Tang	3	2	2	7
Shin	2	1	2	5
Yang	3	2	2	7
Barrett	3	2	2	7
Cao	1	2	2	5
Teng	4	2	2	8
Kwak	2	2	2	6
