# Supplementary material for: Soy Product Consumption and the Risk of Cancer: A Systematic Review and Meta-Analysis of Observational Studies
Source: Nutrients. 2024 Mar 28;16(7):986. doi: 10.3390/nu16070986 (PMC11013307; doi:10.3390/nu16070986)
Supplement: Supplementary file 1 [file nutrients-16-00986-s001.zip › Table S4.pdf]

Supplementary Table S4. Exposure categories and ORs of included studies for this systematic review and meta-analysis.

First author, Year	Exposure category (lowest vs highest)	OR (95% CI)
Chyou,1995	Miso soup, Tofu	
	<1 times/week	1 (ref)
	2-4 times/week	1.41 (0.83-2.40)
	≥5 times/week	1.63 (0.51-5.21)
Galanis,1998	Miso soup (Total)	
	None	1 (ref)
	1 or more times/week	1.2 (0.8-1.8)
	Miso soup (Male)	
	None	1 (ref)
	1 or more times/week	1.2 (0.7-2.0)
	Miso soup (Female)	
	None	1 (ref)
	1 or more times/week	1.3 (0.7-2.4)
Nomura,2004	Tofu	
	0 g/week	1(ref)
	1-60 g/week	1.17 (0.83-1.65)
	61-120 g/week	1.01 (0.74-1.39)
	121-240 g/week	1.18 (0.86-1.63)
	>240 g/week	0.82 (0.54-1.23)
Oba,2007	Soy product (Man)	
	49.22 g/day	1 (ref)
	85.52 g/day	1.12 (0.69-1.84)
	141.09 g/day	1.24 (0.77-2.00)
	Soy product (Woman)	
	46.29 g/day	1 (ref)
	79.60 g/day	0.84 (0.53-1.33)
	128.03 g/day	0.56 (0.34-0.92)
Akhter,2008	Soy food	
	35.4 g/day (Man)	1 (ref)
	65.7 g/day	1.06 (0.83-1.36)
	96.4 g/day	0.94 (0.73-1.22)
	169,9 g/day	0.89 (0.69-1.17)
	35.6 g/day (Woman)	1 (ref)
	65.9 g/day	0.87 (0.64-1.18)
	94.9 g/day	0.90 (0.66-1.23)
	170.3 g/day	1.04 (0.76-1.42)
	Miso Soup	
	147.5 ml/day (Man)	1 (ref)
	260 ml/day	1.05 (0.81-1.35)

	301.7 ml/day	0.98 (0.76-1.27)
	313.7 ml/day	0.88 (0.64-1.10)
	125.6 ml/day (Woman)	1 (ref)
	212.8 ml/day	0.87 (0.64-1.18)
	246.9 ml/day	0.90 (0.66-1.23)
	261.3 ml/day	1.04 (0.76-1.42)
Seow,2009	Soybean drink	
	30.7 g/day	1 (ref)
	66.1 g/day	0.93 (0.69-1.24)
	108.8 g/day	0.77 (0.55-1.06)
	197.7 g/day	0.75 (0.53-1.05)
Wang,2009	Tofu	
	<1 serving/month	1 (ref)
	1-3 serving/month	0.98 (0.83-1.16)
	1 serving/week	0.89 (0.65-1.21)
	≥2 servings/week	0.91 (0.61-1.35)
Yang,2009	Soy foods	
	≤12.8 g/day	1 (ref)
	12.9-21 g/day	0.86 (0.66-1.13)
	>21 g/day	0.67 (0.49-0.90)
Hara,2012	Miso soup	
	63 ml/day(Man)	1 (ref)
	175 ml/day	1.02 (0.83-1.26)
	294 ml/day	1.08 (0.87-1.33)
	449 ml/day	1.17 (0.94-1.47)
	47 ml/day(Woman)	1 (ref)
	140 ml/day	0.85 (0.63-1.14)
	224 ml/day	0.81 (0.59-1.11)
	384 ml/day	0.71 (0.50,1.01)
	Soy food	
	33.4 g/day (Man)	1 (ref)
	59.3 g/day	1.06 (0.87-1.29)
	86.1 g/day	1.03 (0.84-1.26)
	140.6 g/day	1.02 (0.82-1.25)
	33 g/day (Woman)	1 (ref)
	58.7 g/day	0.99 (0.73-1.35)
	85.2 g/day	1.12 (0.83-1.53)
	141 g/d	0.99 (0.71-1.38)
Ollberding,	Tofu	
2012	0-0.21 g/1000kcal/day	1 (ref)
	0.22-0.85 g/1000kcal/day	0.92 (0.71-1.21)
	0.86-2.71 g/1000kcal/day	0.90 (0.68-1.19)
	2.72-7.55 g/1000kcal/day	0.98 (0.70-1.36)
	≥7.56 g/1000kcal/day	0.82 (0.56-1.21)

Ko,2013	Soybean Products (BRCA carries)	
	0-1 times/week	1 (ref)
	2 times/week	1.22 (0.89, 1.67)
	3 times/week	0.98 (0.72, 1.34)
	4-5 times/week	0.69 (0.45, 1.06)
	Soybean Products (BRCA nocarries)	
	0-1 times/week	1 (ref)
	2 times/week	1.05 (0.89,1.23)
	3 times/week	0.96 (0.83,1.12)
	4-5 times/week	0.89 (0.74,1.09)
Wada,2015	Soy foods	
	38.4 g/day (Man)	1 (ref)
	74.1 g/day	0.84 (0.64-1.09)
	102.9 g/day	0.85 (0.65-1.12)
	176.3 g/day	0.71 (0.53-0.96)
	43.5 g/day (Woman)	1 (ref)
	74.9 g/day	1.08 (0.72-1.62)
	102.4 g/day	1.15 (0.76-1.72)
	168.7 g/day	0.58 (0.36-0.94)
	Fermented soy foods	
	6.6 g/day (Man)	1 (ref)
	14.5 g/day	1.13 (0.87-1.47)
	20.5 g/day	0.84 (0.63-1.12)
	37.3 g/day	1.02 (0.77-1.34)
	7.5 g/day (Woman)	1 (ref)
	14.4 g/day	0.95 (0.66-1.36)
	20.9 g/day	0.95 (0.66-1.38)
	34.0 g/day	0.79 (0.52-1.19)
	Nonfermented	
	27.7 g/day (Man)	1 (ref)
	57.3 g/day	0.90 (0.69-1.16)
	80.8 g/day	0.88 (0.67-1.16)
	147.8 g/day	0.68 (0.50-0.91)
	32.0 g/day (Woman)	1 (ref)
	58.1 g/day	0.89 (0.59-1.34)
	80.6 g/day	0.98 (0.66-1.47)
	140.8 g/day	0.63 (0.40-1.00)
Paul,2019	Soy food	
	31.29 g/1000kcal/day	1 (ref)
	64.7 g/1000kcal/day	0.86 (0.66-1.13)
	115.86 g/1000kcal/day	0.80 (0.61-1.05)
Abe,2020	Total soy food	
	Quartile 1 (Man)	1 (ref)
	Quartile 2	1.12 (0.84-1.50)

Quartile 3	1.11 (0.82-1.50)
Quartile 4	1.06 (0.78-1.44)
Quartile 1 (Woman)	1 (ref)
Quartile 2	0.70 (0.42-1.15)
Quartile 3	0.78 (0.48-1.28)
Quartile 4	1.13 (0.71-1.81)
Fermented soy food	
Quartile 1 (Man)	1 (ref)
Quartile 2	0.93 (0.69-1.24)
Quartile 3	0.96 (0.70-1.30)
Quartile 4	0.98 (0.71-1.35)
Quartile 1 (Woman)	1 (ref)
Quartile 2	1.29 (0.79-2.11)
Quartile 3	1.03 (0.61-1.76)
Quartile 4	1.18 (0.68-2.03)
Miso	
Quartile 1 (Man)	1 (ref)
Quartile 2	0.95 (0.64-1.11)
Quartile 3	0.76 (0.57-1.01)
Quartile 4	0.65 (0.48-0.89)
Quartile 1 (Woman)	1 (ref)
Quartile 2	0.86 (0.53-1.40)
Quartile 3	0.90 (0.55-1.47)
Quartile 4	0.98 (0.59-1.62)
Natto	
Quartile 1 (Man)	1 (ref)
Quartile 2	1.14 (0.85-1.53)
Quartile 3	0.92 (0.66-1.29)
Quartile 4	1.33 (0.96-1.83)
Quartile 1 (Woman)	1 (ref)
Quartile 2	0.54 (0.31-0.93)
Quartile 3	0.81 (0.49-1.36)
Quartile 4	0.93 (0.56-1.54)
Unfermented soy food	
Quartile 1 (Man)	1 (ref)
Quartile 2	0.90 (0.67-1.21)
Quartile 3	0.96 (0.72-1.30)
Quartile 4	1.15 (0.86-1.54)
Quartile 1 (Woman)	1 (ref)
Quartile 2	0.82 (0.51-1.33)
Quartile 3	0.64 (0.38-1.08)
Quartile 4	1.27 (0.81-2.00)
Tofu	
Quartile 1 (Man)	1 (ref)

	Quartile 2	0.85 (0.64-1.14)
	Quartile 3	1.00 (0.76-1.33)
	Quartile 4	0.99 (0.74-1.32)
	Quartile 1 (Woman)	1 (ref)
	Quartile 2	0.99 (0.62-1.56)
	Quartile 3	0.81 (0.47-1.31)
	Quartile 4	0.93 (0.57-1.51)
	Soymilk	
	Non-consumer (Man)	1 (ref)
	Consumer	1.34 (0.96-1.88)
	Non-consumer (Man)	1 (ref)
	Consumer	1.28 (0.76-2.16)
Shirabe,2021	Total soy foods	
	31.8 g/day	1 (ref)
	56.4 g/day	1.06 (0.79-1.43)
	82.5 g/day	0.99 (0.73-1.34)
	137 g/day	1.07 (0.79-1.46)
	Fermented soy foods	
	7.3 g/day	1 (ref)
	19.3 g/day	1.25 (0.93-1.69)
	32.6 g/day	1.02 (0.74-1.41)
	53.2 g/day	0.94 (0.67-1.32)
	Miso	
	2.9 g/day	1 (ref)
	9.8 g/day	0.98 (0.73-1.32)
	17.7 g/day	1.03 (0.76-1.40)
	29.1 g/day	0.88 (0.64-1.22)
	Natto	
	0.01 g/day	1 (ref)
	3.8 g/day	1.42 (1.03-1.97)
	11.9 g/day	1.26 (0.90-1.77)
	32.7 g/day	1.08 (0.76-1.54)
	Non-fermented soy foods	
	13.5 g/day	1 (ref)
	28.2 g/day	1.06 (0.79-1.44)
	47.7 g/day	1.18 (0.88-1.58)
	98.5 g/day	1.15 (0.85-1.57)
	Tofu	
	10.3 g/day	1 (ref)
	22.1 g/day	0.98 (0.73-1.33)
	37.3 g/day	1.23 (0.93-1.65)
	74.1 g/day	1.02 (0.75-1.38)
	Soy milk	

	No	1 (ref)
	Yes	1.34 (0.97-1.86)
Nozue, 2021	Total soy product	
	33.6 g/day (Woman)	1 (ref)
	58.7 g/day	0.91 (0.78-1.06)
	85.1 g/day	0.87 (0.75-1.02)
	140.9 g/day	0.92 (0.78-1.07)
	33.7 g/day (Man)	1 (ref)
	59.6 g/day	0.90 (0.80-1.01)
	86.5 g/day	0.97 (0.86-1.09)
	130.9 g/day	1.05 (0.93-1.19)
	Fermented soy products	
	8.6g/day (Woman)	1 (ref)
	21.4 g/day	0.91 (0.78-1.06)
	34.8 g/day	0.86 (0.73-1.01)
	55.1 g/day	0.80 (0.68-0.95)
	9.5 g/day (Man)	1 (ref)
	23.8 g/day	0.97 (0.86-1.10)
	37.7 g/day	1.03 (0.91-1.17)
	60.0 g/day	1.04 (0.91-1.18)
	Nonfermented soy products	
	13.5 g/day (Woman)	1 (ref)
	28.6 g/day	0.87 (0.75-1.02)
	48.5 g/day	0.87 (0.64-1.02)
	99.5 g/day	0.94 (0.80-1.09)
	12.1 g/day (Man)	1 (ref)
	26.4 g/day	0.91 (0.81-1.03)
	46.2 g/day	1.01 (0.90-1.13)
	97 g/day	1.01 (0.89-1.14)
	Miso	
	3.8 g/day (Woman)	1 (ref)
	11.2 g/day	0.92 (0.79-1.07)
	19.9 g/day	0.83 (0.71-0.97)
	30.5 g/day	0.87 (0.74-1.02)
	5.1 g/day (Man)	1 (ref)
	14.0 g/day	1.06 (0.94-1.19)
	23.8 g/day	1.06 (0.94-1.20)
	35.8 g/day	1.09 (0.96-1.23)
	Natto	
	0 g/day (Woman)	1 (ref)
	2.4 g/day	0.95 (0.82-1.12)
	13.4 g/day	0.87 (0.74-1.03)
	32.4 g/day	0.84 (0.71-1.00)
	0.0 g/day	1 (ref)

	3.2 g/day	0.93 (0.83-1.05)
	12.0 g/day	0.91 (0.80-1.04)
	32.1 g/day	0.94 (0.82-1.08)
Shin, 2023	Soybean paste	
	Almost never (Man)	1 (ref)
	≥ 2 times/week	0.81 (0.56-1.16)
	Almost never (Woman)	1 (ref)
	≥ 2 times/week	0.83 (0.6-1.5)
	Tofu	
	Almost never (Man)	1 (ref)
	≥ 2 times/week	0.63 (0.45-0.89)
	Almost never (Woman)	1 (ref)
	≥ 2 times/week	0.93 (0.68-1.26)
Lee,1995	Tofu	
	None or 4-5 times/year	1 (ref)
	1-3 times/week	0.5 (0.1-1.5)
	≥2-3 times/week	0.2 (0.1-0.8)
Horn-Ross,	Tofu	
2001	Nonconsumers	1 (ref)
	<1 times/month	0.79 (0.63-0.99)
	≥1 times/month	0.89 (0.70-1.1)
	Soy milk	
	Nonconsumers	1 (ref)
	Consumers	0.57 (0.38-0.85)
	Miso soup	
	Nonconsumers	1 (ref)
	<1 times/month	1.1 (0.81-1.4)
	≥1 times/month	1.1 (0.81-1.5)
Seow,2002	Soy foods	
	<2.2 servings/week (Smokers)	1 (ref)
	2.2-<5.4 servings/week	1.58 (0.81-3.09)
	≥5.4 servings/week	1.53 (0.76-3.11)
	<2.2 servings/week (Non-smokers)	1 (ref)
	2.2-<5.4 servings/week	0.57 (0.38-0.86)
	≥5.4 servings/week	0.53 (0.34-0.81)
Wu,2002	Tofu	
	Less than monthly	1 (ref)
	1-3 times/month	0.73 (0.47-1.14)
	1-3 times/week	0.62 (0.42-0.92)
	>4 times/week	0.65 (0.38-1.10)
Hirose,2003	Soybean curd (Premenopausal)	
	<1-3 times/month	1 (ref)
	1-2 times/week	0.98 (0.82-1.17)
	3-4 times/week	0.86 (0.71-1.04)

	≥5 times/week	0.84 (0.67-1.04)
	Miso soup	
	Almost never	1 (ref)
	occationally	0.98 (0.72-1.35)
	1 time/day	1.08 (0.79-1.48)
	2 times/day	1.14 (0.79-1.65)
	Soybean curd (Postmenopausal)	
	<1-3 times/month	1 (ref)
	1-2 times/week	0.89 (0.72-1.12)
	3-4 times/week	0.89 (0.70-1.11)
	≥5 times/week	0.83 (0.65-1.05)
	Miso soup	
	Almost never	1 (ref)
	occationally	1.01 (0.72-1.43)
	1 time/day	1.07 (0.76-1.50)
	2 times/day	0.89 (0.61-1.30)
Lee,2003	Soybean curd	
	<1 times/month(ref)	1 (ref)
	≥1 times/month	0.3 (0.2-0.8)
Jian,2004	Fermented soy products	
[67]	0 g/day	1 (ref)
	0.1-4g/day	0.77 (0.41-1.42)
	>4 g/day	2.02 (1.08-3.78)
Hung,2004	Fermented bean product	
	< 1 times/week (20-40 years old)	1 (ref)
	≥1 times/week	1.9 (1.1-3.2)
	< 1 times/week (40+ years old)	1 (ref)
	≥1 times/week	3.4 (1.9-6.2)
Machida-montani, 2004	Miso soup	
	<3 cups/day	1 (ref)
	3 cups/day	1.8 (1.0-3.3)
	≥4 cups/day	2.1 (0.9-5.1)
Sonoda,2004	Tofu	
	≤19.7 g/day	1 (ref)
	19.7-42.7 g/day	0.57 (0.27-1.20)
	42.7-96.4 g/day	0.81 (0.38-1.75)
	≥ 96.4 g/day	0.47 (0.20-1.08)
	All soy products	
	≤7 g/day	1 (ref)
	77-125 g/day	0.75 (0.36-1.63)
	125.1-187.2 g/day	0.66 (0.35-1.26)
	≥187.2 g/day	0.86 (0.37-2.01)
	Natto	
	≤5.7 g/day	1 (ref)

	5.7-13.2 g/day	1.10 (0.53-2.30)
	13.2-40 g/day	0.66 (0.33-1.32)
	≥40 g/day	0.25 (0.05-1.24)
Xu,2004	Soy milk	
	Never	1 (ref)
	≤1.9 g/day	0.90 (0.70-1.17)
	>1.9g/day	0.86 (0.68-1.10)
	Tofu	
	≤0.8 g/day	1 (ref)
	0.9-1.8 g/day	1.10 (0.85-1.44)
	1.9-3.5 g/day	0.90 (0.68-1.21)
	>3.5 g/day	0.80 (0.54-1.19)
	Soya products (no tofu)	
	≤1.8 g/day	1 (ref)
	1.9-5.3 g/day	1.07 (0.81-1.43)
	5.4-8.8 g/day	0.90 (0.68-1.18)
	>8.8 g/day	0.84 (0.59-1.21)
Nan,2005	Soybean paste	
	Low	1 (ref)
	High	1.62 (1.26-2.09)
Hirose,2005	Soybean products	
	17.2 g/1000kcal (Premenopausal)	1 (ref)
	29.7 g/1000kcal	0.60 (0.30-1.18)
	47.9 g/1000kcal	0.53 (0.27-1.04)
	20.1 g/1000kcal (Postmenopausal)	1 (ref)
	35.5 g/1000kcal	0.87 (0.47-1.61)
	56.5 g/1000kcal	0.70 (0.37-1.33)
	Tofu	
	8.9 g/1000kcal (Premenopausal)	1 (ref)
	17.1 g/1000kcal	0.44 (0.22-0.90)
	31.3 g/1000kcal	0.49 (0.25-0.95)
	10.8 g/1000kcal (Postmenopausal)	1 (ref)
	19.6 g/1000kcal	1.34 (0.73-2.44)
	35.9 g/1000kcal	0.71 (0.36-1.39)
	Miso	
	1.4 g/1000kcal (Premenopausal)	1 (ref)
	3.4 g/1000kcal	1.14 (0.59-2.19)
	5.5 g/1000kcal	0.58 (0.28-1.20)
	1.5 g/1000kcal (Postmenopausal)	1 (ref)
	3.9 g/1000kcal	0.52 (0.27-0.98)
	5.8 g/1000kcal	0.64 (0.34-1.17)
	Natto	
	1.2 g/1000kcal (Premenopausal)	1 (ref)
	3.7 g/1000kcal	0.89 (0.46-1.74)

	8.7 g/1000kcal	0.84 (0.43-1.64)
	1.3 g/1000kcal (Postmenopausal)	1 (ref)
	4.0 g/1000kcal	1.00 (0.54-1.87)
	10.8 g/1000kcal	0.79 (0.41-1.51)
Sharp,2005	Miso soup	
	Never or ≤1/week	1 (ref)
	2-4 times/week	0.6 (0.16-2.27)
	≥5 times/week	0.5 (0.14-1.55)
	Tofu	
	Never or ≤1/week	1 (ref)
	2-4 times/week	0.9 (0.31-2.66)
	≥5 times/week	0.9 (0.20-3.51)
Shannon,2005	Total soy food	
	≤2.6 servings/week	1 (ref)
	<4.4 servings/week	0.96 (0.60-1.53)
	<1.1 servings/day	1.33 (0.84-2.12)
	≥1.1 servings/day	1.07 (0.68-1.69)
	Unfermented soy food	
	≤2.3 servings/week	1 (ref)
	<4.2 servinhs/week	1.04 (0.66-1.66)
	<1 servings/d	1.23 (0.77-1.95)
	≥1 servings/day	1.22 (0.78-1.92)
Heald,2007	soy food consumption	
	No (ref)	1 (ref)
	Yes	0.52 (0.30-0.91)
Kim,2008	Tofu	
	<7.73 g/day	1 (ref)
	7.74-14.39 g/day	0.68 (0.39-1.18)
	14.4-23.59 g/day	0.54 (0.31-0.93)
	23.6-49.49 g/day	0.46 (0.27-0.79)
	≥49.5 g/day	0.31 (0.17-0.57)
Li,2008	Soybean food (Tofu and Soymilk)	
	≤2 times/week	1 (ref)
	3-6 times/week	0.38 (0.13-1.12)
	≥1 times/day	0.29 (0.11-0.79)
Matsuo,2008	Soybean products	
	19.8 g/day ($EGFR^{mut}$)	1 (ref)
	44.1 g/day	0.79 (0.50-1.27)
	81.8 g/day	0.56 (0.34-0.93)
	19.8 g/day ($EGFR^{wt}$)	1 (ref)
	44.1 g/day	1.19 (0.85-1.68)
	81.8 g/day	0.89 (0.61-1.29)
	Miso soup	

≤3-4 times/week ($EGFR^{mut}$)	1 (ref)
≤ once a day	0.69 (0.46-1.03)
twice a day	0.23 (0.07-0.77)
≤3-4 times/week ($EGFR^{wt}$)	1 (ref)
≤ once a day	1.08 (0.79-1.48)
twice a day	1.17 (0.70-1.96)
Tofu	
≤1-3 times/month ($EGFR^{mut}$)	1 (ref)
1-2 /week	1.07 (0.66-1.74)
≥3-4 times/week	0.80 (0.48-1.36)
≤1-3 times/month ($EGFR^{wt}$)	1 (ref)
1-2 /week	1.30 (0.92-1.84)
≥3-4 times/week	1.14 (0.78-1.66)
Natto	
≤1-3 times/month ($EGFR^{mut}$)	1 (ref)
≥3-4 times/week	0.63 (0.40-0.99)
≥once a day	0.59 (0.33-1.06)
≤1-3 times/month ($EGFR^{wt}$)	1 (ref)
≥3-4 times/week	0.85 (0.61-1.16)
≥once a day	0.91 (0.58-1.43)
Miso soup (Total)	
≤ 3-4 times/week ($EGFR^{mut}$)	1 (ref)
≤ once a day	0.69 (0.46-1.03)
Twice a day	0.23 (0.07-0.77)
≤ 3-4 times/week ($EGFR^{wt}$)	1 (ref)
≤ once a day	1.08 (0.79-1.48)
Twice a day	1.17 (0.70-1.96)
Miso soup (Male)	
≤ 3-4 times/week ($EGFR^{mut}$)	1 (ref)
≤ once a day	0.46 (0.24-0.91)
Twice a day	0.12 (0.02-0.92)
≤ 3-4 times/week ($EGFR^{wt}$)	1 (ref)
≤ once a day	1.12 (0.78-1.61)
Twice a day	0.90 (0.49-1.68)
Miso soup (Female)	
≤ 3-4 times/week ($EGFR^{mut}$)	1 (ref)
≤ once a day	0.87 (0.52-1.45)
Twice a day	0.35 (0.08-1.57)
≤ 3-4 times/week ($EGFR^{wt}$)	1 (ref)
≤ once a day	1.00 (0.54-1.82)
Twice a day	2.33 (0.89-6.15)
Natto (Total)	
≤ 1-3 times/month ($EGFR^{mut}$)	1 (ref)
≥ 3-4 times/week	0.63 (0.40-0.99)

	≥ once a day	0.59 (0.33-1.06)
	≤ 1-3 times/month ($EGFR^{wt}$)	1 (ref)
	≥ 3-4 times/week	0.85 (0.61-1.16)
	≥ once a day	0.91 (0.58-1.43)
	Natto (Male)	
	≤ 1-3 times/month ($EGFR^{mut}$)	1 (ref)
	≥ 3-4 times/week	0.57 (0.28-1.17)
	≥ once a day	0.56 (0.22-1.43)
	≤ 1-3 times/month ($EGFR^{wt}$)	1 (ref)
	≥ 3-4 times/week	0.89 (0.61-1.29)
	≥ once a day	0.84 (0.48-1.47)
	Natto (Female)	
	≤ 1-3 times/month ($EGFR^{mut}$)	1 (ref)
	≥ 3-4 times/week	0.69 (0.38-1.22)
	≥ once a day	0.62 (0.29-1.34)
	≤ 1-3 times/month ($EGFR^{wt}$)	1 (ref)
	≥ 3-4 times/week	0.71 (0.38-1.35)
	≥ once a day	1.01 (0.45-2.29)
	Tofu (Total)	
	≤ 1-3 times/month ($EGFR^{mut}$)	1 (ref)
	1-2 times/week	1.07 (0.66-1.74)
	≥ 3-4 times/week	0.80 (0.48-1.36)
	≤ 1-3 times/month ($EGFR^{wt}$)	1 (ref)
	1-2 times/week	1.30 (0.92-1.84)
	≥ 3-4 times/week	1.14 (0.78-1.66)
	Tofu (Male)	
	≤ 1-3 times/month ($EGFR^{mut}$)	1 (ref)
	1-2 times/week	0.54 (0.25-1.15)
	≥ 3-4 times/week	0.61 (0.27-1.39)
	≤ 1-3 times/month ($EGFR^{wt}$)	1 (ref)
	1-2 times/week	1.33 (0.90-1.97)
	≥ 3-4 times/week	0.97 (0.61-1.53)
	Tofu (Female)	
	≤ 1-3 times/month ($EGFR^{mut}$)	1 (ref)
	1-2 times/week	1.85 (0.93-3.69)
	≥ 3-4 times/week	1.15 (0.55-2.38)
	≤ 1-3 times/month ($EGFR^{wt}$)	1 (ref)
	1-2 times/week	1.06 (0.51-2.23)
	≥ 3-4 times/week	1.46 (0.73-2.94)
Bandera,2009	Tofu	
	Never	1 (ref)
	Ever	0.68 (0.45-1.03)
	Soy milk	
	Never	1 (ref)

	Ever	0.85 (0.55-1.33)
	Miso	
	Never	1 (ref)
	Ever	1.31 (0.83-2.07)
Liu,2009	Bean-curd food	
	Rare or occasional (2-5 years old)	1 (ref)
	Frequent	0.47 (0.22-0.99)
	Rare or occasional (2-20 years old)	1 (ref)
	Frequent	0.55 (0.34-0.89)
Hosono,2010	Tofu	
	None (invasive carcinoma)	1 (ref)
	<1 times/week	0.80 (0.49-1.32)
	1-4 times/week	0.78 (0.48-1.26)
	>5 times/week	0.64 (0.33-1.25)
	None (CIN3)	1 (ref)
	<1 times/week	1.45 (0.49-4.30)
	1-4 times/week	0.76 (0.26-2.23)
	>5 times/week	0.55 (0.13-2.36)
Budhathoki	Soy foods (Total)	
2011	5.4 g/day	1 (ref)
	8.8 g/day	0.89 (0.65-1.22)
	13.1 g/day	0.82 (0.59-1.13)
	18.5 g/day	0.66 (0.47-0.94)
	26.8 g/day	0.73 (0.51-1.04)
	Soy foods (Men)	
	5.4 g/day	1 (ref)
	8.8 g/day	0.80 (0.55-1.17)
	13.1 g/day	0.80 (0.53-1.19)
	18.5 g/day	0.55 (0.34-0.88)
	26.8 g/day	0.65 (0.41-1.03)
	Soy foods (Women)	
	5.4 g/day	1 (ref)
	8.8 g/day	1.11 (0.61-2.04)
	13.1 g/day	0.89 (0.50-1.60)
	18.5 g/day	0.90 (0.51-1.61)
	26.8 g/day	0.92 (0.50-1.67)
Chihara,2012	Miso soup	
	<1 time/week (Man)	1 (ref)
	1-6 times/week	1.21 (0.58-2.55)
	≥1 time/day	1.34 (0.63-2.83)
	<1 time/week (Woman)	1 (ref)
	1-6 times/week	0.93 (0.49-1.79)
	≥1 time/day	0.75 (0.37-1.53)
	Tofu	

	<1 time/week (Man)	1 (ref)
	1-6 times/week	1.13 (0.77-1.66)
	≥1 time/day	1.60 (0.70-3.67)
	<1 time/week (Woman)	1 (ref)
	1-6 times/week	0.98 (0.65-1.47)
	≥1 time/day	1.09 (0.47-2.55)
	Natto	
	<1 time/week (Man)	1 (ref)
	1-6 times/week	1.01 (0.68-1.50)
	≥1 time/day	0.83 (0.41-1.69)
	<1 time/week (Woman)	1 (ref)
	1-6 times/week	0.59 (0.39-0.88)
	≥1 time/day	0.68 (0.35-1.31)
Wang,2012	Soy food	
	Never	1 (ref)
	1-3 times/month	0.30 (0.03-2.99)
	1-2 times/week	0.17 (0.02-1.63)
	≥3 times/week	0.10 (0.01-0.94)
Wang,2012	Soya products	
	Tertile 1	1 (ref)
	Tertile 2	0.2 (0.07-2.3)
	Tertile 3	0.04 (0.01-0.3)
Zaineddin,	Soy milk	
2012	No consumption	1 (ref)
	Low consumption	0.95 (0.73-1.25)
	High consumption	0.94 (0.75-1.18)
	Tofu	
	No consumption	1 (ref)
	Low consumption	0.99 (0.76-1.28)
	High consumption	0.89 (0.73-1.07)
	Paste	
	No consumption	1 (ref)
	Low consumption	0.91 (0.71-1.16)
	High consumption	0.79 (0.62-1.03)
Lee,2014	Total soy foods	
	≤61.4 g/day	1 (ref)
	61.5-119 g/day	0.63 (0.46-0.86)
	>119 g/day	0.29 (0.20-0.42)
	Soy milk	
	≤12.9 ml/day	1 (ref)
	13-38.6 ml/day	0.66 (0.47-0.91)
	>38.6 ml/day	0.43 (0.31-0.60)
	Tofu	
	≤8.6 g/day	1 (ref)

	8.7-20 g/day	1.00 (0.73-1.36)
	>20 g/day	0.57 (0.40-0.80)
Tang,2015	Total soya foods	
	<26 g/day	1 (ref)
	26-97 g/day	0.53 (0.37-0.76)
	>97 g/day	0.33 (0.22-0.49)
	Soya milk	
	<2 ml/day	1 (ref)
	2-60 ml/day	0.58 (0.40-0.86)
	>60 ml/day	0.48 (0.31-0.73)
Shin,2015	Soy products	
	<40.34 g/day (Man)	1 (ref)
	40.34-<64.64 g/day	1.38 (1.05-1.80)
	64.64-105.03 g/day	1.40 (1.07-1.83)
	≥105.03 g/day	0.67 (0.49-0.92)
	<42.77 g/day (Woman)	1 (ref)
	42.77-<70.29 g/day	1.27 (0.86-1.88)
	70.29-113.66 g/day	1.37 (0.92-2.04)
	≥113.66 g/day	0.62 (0.39-1.00)
	Tofu	
	<17.19 g/day (Man)	1 (ref)
	17.19-<30.77 g/day	1.82 (1.37-2.42)
	30.77-52.86 g/day	2.03 (1.54-2.69)
	≥52.86 g/day	0.94 (0.68-1.29)
	<18.73 g/day (Woman)	1 (ref)
	18.73-32.68 g/day	1.80 (1.19-2.72)
	32.68-<54.91 g/day	1.80 (1.20-2.72)
	≥54.91 g/day	0.97 (0.61-1.52)
	Soymilk	
	0 g/day (Man)	1 (ref)
	0-<21.35 g/day	1.74 (1.39-2.17)
	≥21.35g/day	0.47 (0.34-0.64)
	0 g/day (Woman)	1 (ref)
	0-<19.1 g/day	1.50 (1.04-2.15)
	≥19.1g/day	0.82 (0.54-1.25)
	Fermented soy paste	
	<1.95 g/day (Woman)	1 (ref)
	1.95-<4.04 g/day	1.81 (1.33-2.45)
	4.04-<8.32 g/day	2.06 (1.53-2.78)
	≥8.32 g/day	1.82 (1.35-2.46)
	<2.08 g/day (Woman)	1 (ref)
	2.08-<4.5 g/day	1.62 (1.06-2.49)
	4.5-<8.7 g/day	1.57 (1.02-2.41)
	≥8.7 g/day	1.22 (0.77-1.91)

Yang,2017	Total soy products (Total)	
	≤48.39 g/day	1 (ref)
	48.39-86.2 g/day	1.23 (0.88-1.72)
	>86.2 g/day	0.85 (0.59-1.20)
	Total soy products (Male)	
	≤48.39 g/day	1 (ref)
	48.39-86.2 g/day	1.34 (0.89-2.02)
	>86.2 g/day	0.69 (0.43-1.09)
	Total soy products (Female)	
	≤48.39 g/day	1 (ref)
	48.39-86.2 g/day	0.98 (0.54-1.79)
	>86.2 g/day	1.07 (0.61-1.89)
	Fermented soy foods (Total)	
	≤2.29 g/day	1 (ref)
	2.29-5.78 g/day	0.97 (0.68-1.37)
	>5.78 g/day	1.08 (0.77-1.51)
	Fermented soy foods (Male)	
	≤2.29 g/day	1 (ref)
	2.29-5.78 g/day	0.93 (0.60-1.43)
	>5.78 g/day	0.90 (0.59-1.38)
	Fermented soy foods (Female)	
	≤2.29 g/day	1 (ref)
	2.29-5.78 g/day	1.06 (0.58-1.94)
	>5.78 g/day	1.46 (0.89-2.63)
	Nonfermented soy foods (Total)	
	≤42.95 g/day	1 (ref)
	42.95-85.54 g/day	1.13 (0.81-1.56)
	>85.54 g/day	0.62 (0.43-0.90)
	Nonfermented soy foods (Male)	
	≤42.95 g/day	1 (ref)
	42.95-85.54 g/day	1.14 (0.76-1.71)
	>85.54 g/day	0.44 (0.27-0.71)
	Nonfermented soy foods (Female)	
	≤42.95 g/day	1 (ref)
	42.95-85.54 g/day	1.07 (0.60-1.92)
	>85.54 g/day	0.97 (0.54-1.77)
	Tofu (Total)	
	≤20.47 g/day	1 (ref)
	20.47-40.05 g/day	0.79 (0.56-1.12)
	>40.05 g/day	0.91 (0.65-1.28)
	Tofu (Male)	
	≤20.47 g/day	1 (ref)
	20.47-40.05 g/day	0.86 (0.56-1.31)
	>40.05 g/day	0.79 (0.52-1.21)

	Tofu (Female)	
	≤20.47 g/day	1 (ref)
	20.47-40.05 g/day	0.66 (0.63-1.21)
	>40.05 g/day	1.12 (0.53-1.99)
	Soymilk (Total)	
	≤4.24E^{-9} g/day	1 (ref)
	4.24E^{-9}-3.55 g/day	0.69 (0.49-0.96)
	≥3.55 g/day	0.61 (0.43-0.87)
	Soymilk (Male)	
	≤4.24E^{-9} g/day	1 (ref)
	4.24E^{-9}-3.55 g/day	0.74 (0.49-1.12)
	≥3.55 g/day	0.60 (0.39-0.92)
	Soymilk (Female)	
	≤4.24E^{-9} g/day	1 (ref)
	4.24E^{-9}-3.55 g/day	0.60 (0.34-1.08)
	≥3.55 g/day	0.63 (0.35-1.13)
Barrett,2019	Bean paste (Adult)	
	0 g/day (Men)	1 (ref)
	>0-<0.61 g/day	0.69 (0.58-0.82)
	0.61-<1.66 g/day	0.72 (0.60-0.85)
	≥1.66 g/day	0.67 (0.56-0.80)
	0 g/day (Woman)	1 (ref)
	>0-≤0.54 g/day	0.69 (0.58-0.82)
	>0.54-<1.66 g/day	0.72 (0.60-0.85)
	≥1.66 g/day	0.67 (0.56-0.80)
	Bean paste (Adolescent)	
	0 g/day	1 (ref)
	>0-<0.66 g/day	0.83 (0.70-0.99)
	0.66-2.5 g/day	0.75 (0.63-0.90)
	>2.5 g/day	0.78 (0.65-0.94)
Cao,2022	Soy foods	
	0-3.3 g/day	1 (ref)
	3.4-28.6 g/day	0.68 (0.51-0.91)
	28.6-57.1 g/day	0.65 (0.50-0.86)
	≥57.1 g/day	0.52 (0.39-0.69)
Teng,2023	Soybean products (Total)	
	0-10 g/day	1 (ref)
	10.1-21.7 g/day	0.69 (0.35-1.36)
	21.8-41.7 g/day	0.60 (0.33-1.08)
	41.8-181.7 g/day	0.75 (0.40-1.41)
	Soybean products (Male)	
	0-10 g/day	1 (ref)
	10.1-23.3 g/day	1.18 (0.51-2.73)
	23.4-41.7 g/day	0.95 (0.46-1.96)

	41.8-180 g/day	1.23 (0.55-2.71)
	Soybean products (Female)	
	0-10 g/day	1 (ref)
	10.1-20 g/day	0.31 (0.09-1.06)
	20.1-41.7 g/day	0.25 (0.08-0.78)
	41.8-181.7 g/day	0.33 (0.11-1.01)
Kwak,2023	Soy products	
	Tertile 1	1 (ref)
	Tertile 2	0.38 (0.15-0.92)
	Tertile3	0.37 (0.14-0.96)

[1] ref, reference; CI, confidence interval
